# The effect of smoking on COVID‐19 severity: A systematic review and meta‐analysis

**DOI:** 10.1002/jmv.26389

**Published:** 2020-08-13

**Authors:** Rohin K. Reddy, Walton N. Charles, Alexandros Sklavounos, Atul Dutt, Paul T. Seed, Ankur Khajuria

**Affiliations:** ^1^ Department of Surgery and Cancer Imperial College London London UK; ^2^ Department of Women and Children's Health King's College London London UK; ^3^ Kellogg College University of Oxford Oxford UK

**Keywords:** coronavirus, epidemiology, pandemics, pathogenesis, respiratory tract, virus classification, zoonoses

## Abstract

Various comorbidities represent risk factors for severe coronavirus disease 2019 (COVID‐19). The impact of smoking on COVID‐19 severity has been previously reported in several meta‐analyses limited by small sample sizes and poor methodology. We aimed to rigorously and definitively quantify the effects of smoking on COVID‐19 severity. MEDLINE, Embase, CENTRAL, and Web of Science were searched between 1 December 2019 and 2 June 2020. Studies reporting smoking status of hospitalized patients with different severities of disease and/or at least one clinical endpoint of interest (disease progression, intensive care unit admission, need for mechanical ventilation, and mortality) were included. Data were pooled using a random‐effects model. This study was registered on PROSPERO: CRD42020180920. We analyzed 47 eligible studies reporting on 32 849 hospitalized COVID‐19 patients, with 8417 (25.6%) reporting a smoking history, comprising 1501 current smokers, 5676 former smokers, and 1240 unspecified smokers. Current smokers had an increased risk of severe COVID‐19 (risk ratios [RR]: 1.80; 95% confidence interval [CI]: 1.14‐2.85; *P* = .012), and severe or critical COVID‐19 (RR: 1.98; CI: 1.16‐3.38; *P* = .012). Patients with a smoking history had a significantly increased risk of severe COVID‐19 (RR: 1.31; CI: 1.12‐1.54; *P* = .001), severe or critical COVID‐19 (RR: 1.35; CI: 1.19‐1.53; *P* < .0001), in‐hospital mortality (RR: 1.26; CI: 1.20‐1.32; *P* < .0001), disease progression (RR: 2.18; CI: 1.06‐4.49; *P* = .035), and need for mechanical ventilation (RR: 1.20; CI: 1.01‐1.42; *P* = .043). Patients with any smoking history are vulnerable to severe COVID‐19 and worse in‐hospital outcomes. In the absence of current targeted therapies, preventative, and supportive strategies to reduce morbidity and mortality in current and former smokers are crucial.

## Highlights

- The first high‐quality systematic review and meta‐analysis assessing the impact of smoking on COVID‐19 severity.
- To date, the largest meta‐analysis among peer‐reviewed literature assessing the impact of smoking on COVID‐19 severity, including 32,849 hospitalised patients with COVID‐19.
- Patients who were current smokers had an increased risk of severe COVID‐19 and severe or critical COVID‐19.
- Patients with a smoking history had an increased risk of severe COVID‐19, severe or critical COVID‐19, in‐hospital mortality, disease progression and need for mechanical ventilation.

## 1 INTRODUCTION

As of 28 July 2020, severe acute respiratory syndrome coronavirus 2 (SARS‐CoV‐2) has infected 16 341 920 patients, with 650 805 deaths across 188 countries.^1^, ^2^ Risk factors for poor outcome in patients with coronavirus disease 2019 (COVID‐19) include older age, male sex, hypertension, diabetes, cardiovascular disease, and respiratory disease.^3^, ^4^, ^5^ Remarkably, current peer‐reviewed data surrounding the effect of smoking tobacco on the clinical severity of COVID‐19 has thus far been controversial, and there is an urgent need for definitive answers.^6^

An early systematic review without meta‐analysis concluded that smoking is most likely associated with negative progression and outcomes in COVID‐19,^7^ however, a preliminary meta‐analysis showed that active smoking is not significantly associated with increased risk of severe disease.^8^ Four subsequent meta‐analyses have shown an increased risk of severe COVID‐19 associated with smoking.^9^, ^10^, ^11^, ^12^ A summary of the six previously published systematic reviews^7^, ^8^, ^9^, ^10^, ^11^, ^12^ alongside assessment of their methodological quality using A Measurement Tool to Assess systematic Reviews 2^13^ (AMSTAR 2) is provided in the Appendix (Appendix pp2‐3). The articles ranged from critically poor to moderate quality, indicating that significant methodological flaws in critical domains exist with all six currently published reviews assessing the impact of smoking on COVID‐19 severity. It is therefore likely that the true effect of smoking on COVID‐19 severity reported in these analyses is clouded by considerable bias.

Furthermore, as a result of several nonpeer reviewed preprint articles falsely equating the prevalence of smoking in COVID‐19 study populations with population estimates for smoking prevalence, there has been widespread attention paid to recent mass media reports that smoking may exert a protective effect against COVID‐19 infection.^14^ This led to the World Health Organization releasing a statement on 11 May urging caution with regards to these claims, and emphasizing the lack of evidence confirming a link between smoking or nicotine in the prevention or treatment of COVID‐19.^15^ Consequently, there remains a distinct lack of clarity and high‐quality evidence regarding the relationship between smoking and the severity of COVID‐19. Therefore, to address this important clinical question, this systematic review and meta‐analysis aimed to evaluate the effect of smoking status, including current smoking and a history of smoking, on the clinical severity of COVID‐19.

## 2 METHODS

### 2.1 Search strategy and selection criteria

This systematic review and meta‐analysis adhered to PRISMA guidelines^16^ and was AMSTAR 2 compliant (Appendix pp8‐12).^13^ Two authors independently searched MEDLINE, Embase, CENTRAL, and Web of Science for studies published between 1 December 2019 and 2 June 2020. The search strategy is provided in the Appendix (p13). No language restrictions were applied. COVID‐19 resource centers of *The Lancet, The Lancet Respiratory Medicine, The New England Journal of Medicine*, and *The BMJ* were also hand searched up to 5 July 2020. Reference lists of included studies and previous systematic reviews were additionally screened for their relevance.

To capture all available relevant evidence, randomized, and observational studies reporting the smoking status of hospitalized patients presenting with different severities of disease and/or at least one clinical endpoint of interest were deemed eligible for inclusion. Smoking history included current and former tobacco smokers or e‐cigarette users. Disease severity, including severe or critical cases, was defined a priori and based on the COVID‐19 diagnostic criteria issued by the Chinese National Health Commission (Appendix p13).^17^ Other acceptable criteria included the Infectious Diseases Society of America/American Thoracic Society (IDSA/ATS) criteria for severe community‐acquired pneumonia.^18^ Clinical endpoints of disease progression, intensive care unit (ICU) admission, mechanical ventilation requirement, and/or mortality were used as surrogate markers for in‐hospital severity. We excluded studies on other coronaviruses or if there was insufficient information to distinguish disease severity based on smoking status. Case series involving less than 20 patients, review articles, editorials, conference abstracts, and nonclinical studies were also excluded. Preprints were not assessed for eligibility due to their preliminary nature.

Two authors (WNC, AS) independently screened the titles and abstracts of retrieved studies, with full‐texts of all potentially eligible papers subsequently assessed for inclusion. Any discrepancy was resolved by consensus discussion with the senior author (AK).

### 2.2 Data analysis

Data from studies that fulfilled our inclusion criteria were extracted independently by three authors (WNC, AS, and RKR). Main data‐points included: study details (author, journal, date, country, study design, study period, and funding), total numbers of patients, and their clinical outcomes by smoking status.

Two authors (AS and AD) independently assessed the quality of included studies using the Newcastle‐Ottawa Scale modified for case series, cohort studies, and cross‐sectional studies.^19^ Scores were then classified by the Agency for Healthcare Research and Quality standards as good, fair, or poor. Any discrepancies in quality assessment were resolved by a third author (WNC).

As per our prespecified analysis plan, random‐effects meta‐analyses of pooled raw data were employed using the DerSimonian and Laird method for each outcome with sufficient data to account for anticipated differences across countries and study design over time. Current smokers were compared to former and never‐smokers, and patients with a smoking history were compared to never‐smokers. Where available, adjusted effect estimates were combined and in the absence of adjustment for confounders, raw effect estimates were combined. The results are presented in forest plots as risk ratios (RR) and corresponding 95% confidence intervals (CI) for each outcome. *I* ^2^ estimates of heterogeneity, representing the variability across studies, are classified as low (<30%), moderate (30%‐60%), or high (>60%). Sensitivity analyses included only good‐quality studies and, for severity outcomes, studies using the COVID‐19‐specific criteria for grading severity. Subgroup analyses were completed by country. Funnel plots were used to check for publication bias and tested for asymmetry using Harbord's test,^20^ with studies with no events in either exposed or unexposed arms excluded from this analysis. *P* values <.05 were considered significant.

Data were analyzed using Stata (version 15). The study protocol was prospectively registered with PROSPERO, number CRD42020180920.^21^

### 2.3 Role of the funding source

This study received no funding. All authors had full access to all of the data and took responsibility for the decision to submit for publication.

## 3 RESULTS

**Figure 1:**
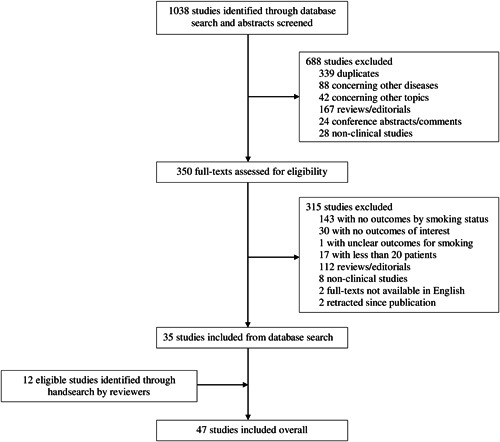
Flow diagram of selection of included studies

The search identified 1038 papers, of which 339 were duplicates. After screening the titles and abstracts of the remaining 699 papers, 350 full‐texts were reviewed. Overall, 35 studies met the inclusion criteria, with a further 12 identified from the references of included studies or by the reviewer team (Figure 1). The 47 included studies^4^, ^22^, ^23^, ^24^, ^25^, ^26^, ^27^, ^28^, ^29^, ^30^, ^31^, ^32^, ^33^, ^34^, ^35^, ^36^, ^37^, ^38^, ^39^, ^40^, ^41^, ^42^, ^43^, ^44^, ^45^, ^46^, ^47^, ^48^, ^49^, ^50^, ^51^, ^52^, ^53^, ^54^, ^55^, ^56^, ^57^, ^58^, ^59^, ^60^, ^61^, ^62^, ^63^, ^64^, ^65^, ^66^, ^67^ represented a total of 32 849 hospitalized COVID‐19 patients: 8417 (25.6%) with any reported smoking history, comprising 1501 current smokers, 5676 former smokers, and 1240 unspecified smokers; 22 420 (68.3%) never‐smokers; and a further 2012 (6.1%) patients who did not currently smoke, though it was unclear whether they were former or never‐smokers (Table 1).

**Table 1:** Characteristics of included studies

	Setting	Study design	Number of centers	Study period	Number of patients, current smokers vs former/never‐smokers	Number of patients, any smoking history vs never‐smokers	Study quality
Azar et al^22^	United States	Cohort	24	Jan‐Apr	10 vs 216	73 vs 153	Fair
Bhargava et al^23^	United States	Cohort	1	Mar‐Apr	11 vs 186	⋯	Good
Bi et al^24^	China	Cohort	1	Jan‐Mar	8 vs 105	⋯	Good
Brenner et al^25^	International	Cohort	1+	‐Apr	11 vs 150	⋯	Poor
Buckner et al^26^	United States	Case series	3	Mar‐May	⋯	22 vs 64	Poor
CDC COVID‐19 Response Team^27^	United States	Cohort	1+	Feb‐Mar	27 vs 1467	105 vs 1389	Poor
Chen et al^28^	China	Case series	1	Jan‐Mar	⋯	15 vs 130	Poor
Chen et al^29^	China	Cohort	575	‐Jan	⋯	111 vs 1479	Good
Chen et al^30^	China	Case series	1	Jan‐Feb	12 vs 262	⋯	Poor
Docherty et al^31^	UK	Cohort^‡^	208	Feb‐May	852 vs 13 332	5216 vs 8968	Good
Feng et al^32^	China	Cohort	3	Jan‐Mar	⋯	44 vs 410	Good
Goyal et al^33^	United States	Case series	2	Mar‐Apr	20 vs 373	98 vs 295	Poor
Guan et al^34^	China	Cohort	552	Dec‐Jan	137 vs 948	158 vs 927	Poor
Hu et al^35^	China	Case series	1	Jan‐Mar	⋯	38 vs 285	Good
Huang et al^36^	China	Case series^‡^	1	Dec‐Jan	3 vs 38	⋯	Poor
Huang et al^37^	China	Cohort	1	Jan‐Mar	56 vs 288	⋯	Good
Huang et al^38^	China	Case series	8	Jan‐Feb	⋯	16 vs 186	Good
Hur et al^39^	United States	Cohort	10	Mar‐Apr	16 vs 470	163 vs 323	Good
Inciardi et al^40^	Italy	Cohort	1	Mar‐Mar	⋯	17 vs 82	Poor
Ji et al^41^	China	Cohort	2	Jan‐Mar	⋯	19 vs 189	Good
Kalligeros et al^42^	United States	Cohort	3	Feb‐Apr	12 vs 91	48 vs 55	Good
Klang et al^43^	United States	Cohort	5	Mar‐May	⋯	793 vs 2613	Good
Kuderer et al^44^	International^a^	Cohort	1+	Mar‐May	25 vs 406	226 vs 205	Fair
Li et al^45^	China	Cohort^†^	1	Jan‐Mar	41 vs 503	92 vs 452	Good
Li et al^46^	China	Case series	1	Jan‐Feb	⋯	7 vs 18	Poor
Liu et al^47^	China	Cohort	3	Dec‐Jan	⋯	5 vs 73	Good
Petrilli et al^49^	United States	Cohort^‡^	4	Mar‐May	141 vs 2145	702 vs 1584	Good
Qin et al^48^	China	Cohort	1	Jan‐Feb	⋯	7 vs 445	Poor
Rastrelli et al^50^	Italy	Case series	1	⋯	1 vs 30	12 vs 19	Poor
Shi et al^51^	China	Cohort	2	Jan‐Mar	⋯	16 vs 290	Good
Shi et al^52^	China	Cohort	1+	‐Feb	⋯	40 vs 434	Good
Sun et al^53^	China	Cohort	1	Feb‐Mar	⋯	12 vs 45	Good
Toussie et al^54^	United States	Cohort	1+	Mar‐Mar	⋯	29 vs 94	Fair
Wan et al^55^	China	Case series^‡^	1	Jan‐Feb	9 vs 126	⋯	Poor
Wang et al^56^	China	Cohort^†^	1	⋯	41 vs 503	92 vs 452	Poor
Wang et al^57^	China	Cohort	1	Jan‐Feb	16 vs 109	16 vs 109	Poor
Yang et al^58^	China	Cohort	1	Dec‐Feb	⋯	2 vs 50	Poor
Yao et al^59^	China	Cohort	1	Jan‐Mar	4 vs 104	⋯	Good
Yu et al^60^	China	Cohort	24	Jan‐Mar	13 vs 408	⋯	Good
Yu et al^61^	China	Cross‐sectional	2	Jan‐Feb	⋯	5 vs 65	Good
Yu et al^62^	China	Cohort	1	Jan‐Mar	⋯	16 vs 76	Poor
Yu et al^63^	China	Cohort	1+	Dec‐Feb	⋯	26 vs 265	Fair
Zhang et al^64^	China	Case series	1	Jan‐Feb	2 vs 138	9 vs 131	Poor
Zhang et al^65^	China	Cohort	1	Jan‐Feb	6 vs 114	⋯	Fair
Zheng et al^66^	China	Cohort^‡^	3	Jan‐Feb	8 vs 58	⋯	Fair
Zheng et al^67^	China	Case series	1	Jan‐Feb	8 vs 65	8 vs 65	Poor
Zhou et al^4^	China	Cohort	2	Dec‐Jan	11 vs 180	⋯	Good

*Note*: All studies are retrospective except: ^†^ambispective (includes prospective and retrospective components) and ^‡^prospective.

a Contains data from the United States, Canada, and Spain.

There were 25 multicentre studies (three prospective^31^, ^49^, ^66^ and 22 retrospective)^4^, ^22^, ^25^, ^26^, ^27^, ^29^, ^32^, ^33^, ^34^, ^38^, ^39^, ^41^, ^42^, ^43^, ^44^, ^47^, ^51^, ^52^, ^54^, ^60^, ^61^, ^63^ and 22 single‐centre studies (two prospective,^36^, ^55^ two with prospective and retrospective components,^45^, ^56^ and 18 retrospective^23^, ^24^, ^28^, ^30^, ^35^, ^37^, ^40^, ^46^, ^48^, ^50^, ^53^, ^57^, ^58^, ^59^, ^62^, ^64^, ^65^, ^67^). The majority of studies investigated a Chinese population (32/47, 68%), with the United States contributing 10 studies. Overall, study quality was good in 22 studies, fair in six and poor in 19 (Appendix p17). Of 38 studies disclosing funding status, 28 received funding.

Three studies^32^, ^56^, ^64^ reported smoking index or pack‐years by outcome of interest. Six studies^23^, ^25^, ^39^, ^49^, ^54^, ^61^ reported outcomes for tobacco smokers, including one^25^ that had pooled outcomes with those of e‐cigarette users. The remaining studies did not specify the substance of smoking.

Disease severity was graded according to the Chinese COVID‐19‐specific criteria in 14 studies,^24^, ^28^, ^32^, ^35^, ^38^, ^46^, ^48^, ^53^, ^55^, ^57^, ^63^, ^64^, ^65^, ^66^ the IDSA/ATS criteria in three studies^34^, ^45^, ^59^ and a locally devised criteria in one study (Appendix p13).^54^ Two studies^52^, ^62^ did not specify the criteria utilized.

**Figure 2:**
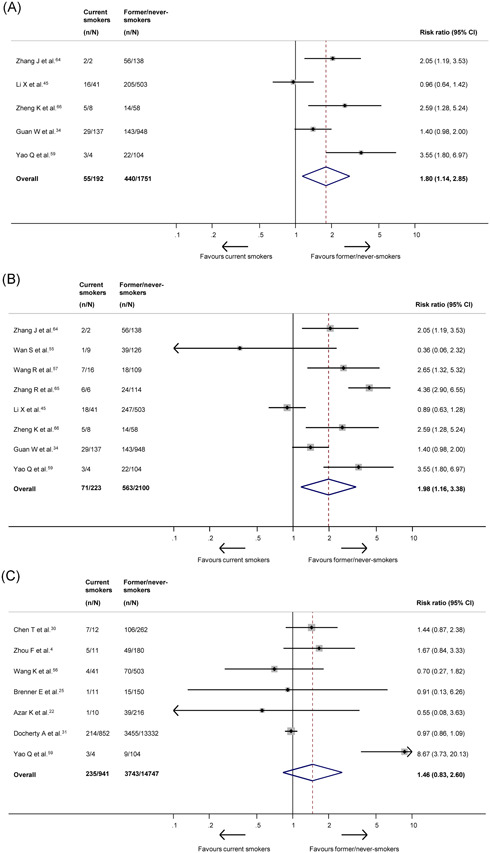
A, Forest plot showing the effect of current smoking on severe COVID‐19. B, Forest plot showing the effect of current smoking on severe or critical COVID‐19. C, Forest plot showing the effect of current smoking on mortality. COVID‐19, coronavirus disease 2019

Current smokers, whose outcomes were evaluated in 27 studies, had an overall prevalence of 6.2% (specifically, China: 8.7%, United States: 4.6%). They had a significantly increased risk of presenting with severe disease (RR: 1.80; 95% CI: 1.14‐2.85; *P* = .012; *I* ^2^ = 76%; Figure 2A), as well as severe or critical disease (RR: 1.98; 95% CI: 1.16‐3.38; *P* = .012; *I* ^2^ = 87%; Figure 2B), compared to former or never‐smokers. Effects were consistent when only analyzing studies using the COVID‐19‐specific criteria (Appendix p22). On sensitivity analysis, including only good‐quality studies resulted in these effects becoming nonsignificant. There were no significant effects on in‐hospital outcomes, including disease progression (RR: 1.54; 95% CI: 0.52‐4.58; *P* = .439; *I* ^2^ = 81%; Appendix p19), ICU admission (RR: 0.72; 95% CI: 0.42‐1.24; *P* = .237; *I* ^2^ = 40%; Appendix p20), mechanical ventilation requirement (RR: 1.13; 95% CI: 0.75‐1.72; *P* = .561; *I* ^2^ = 32%; Appendix p21) or mortality (RR: 1.46; 95% CI: 0.83‐2.60; *P* = .192; *I* ^2^ = 81%; Figure 2C). There were no differences in outcomes by country of origin (Appendix p23). A meta‐analysis was not performed for critical disease alone as only one study reported this outcome.

**Figure 3:**
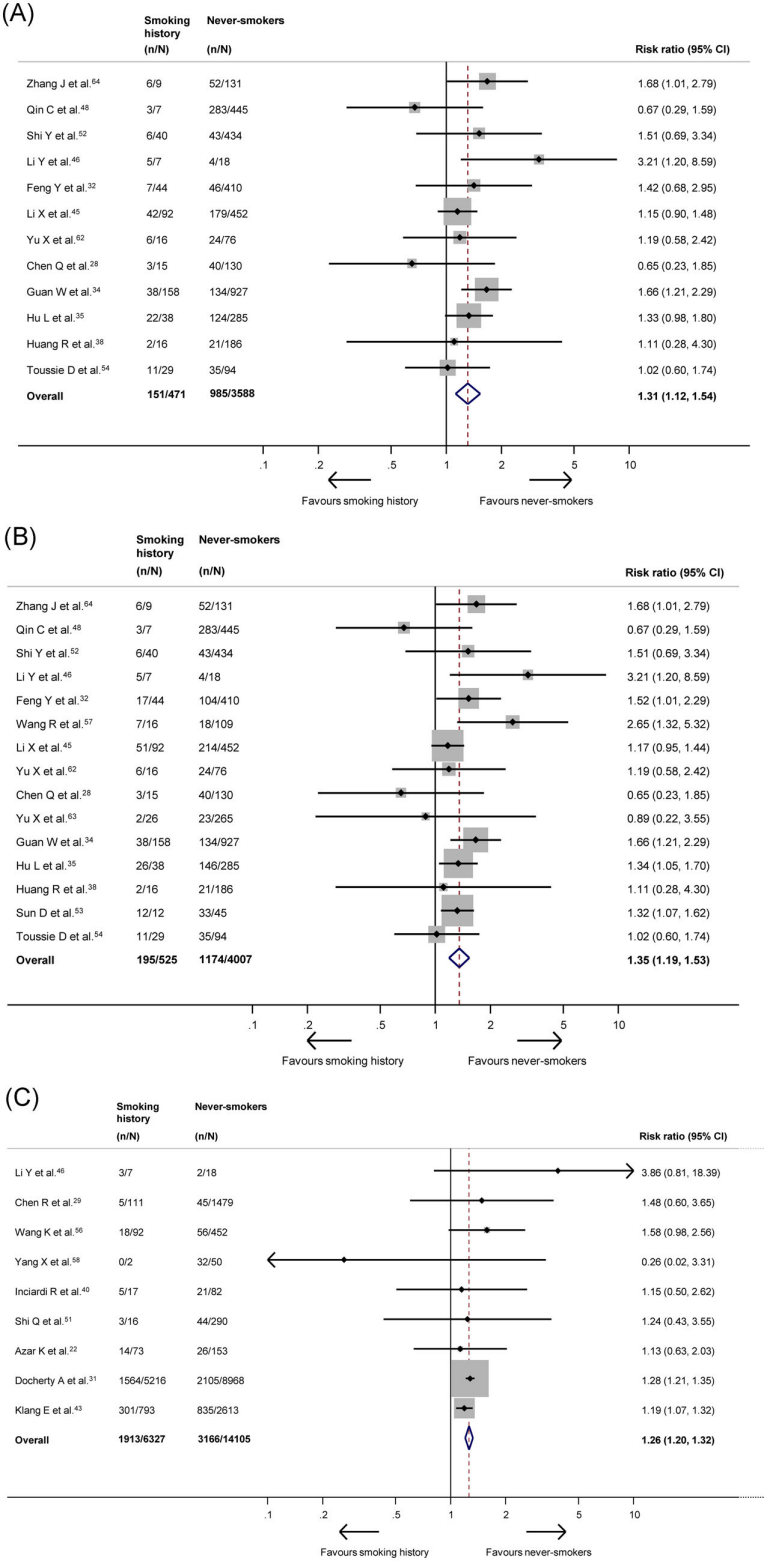
A, Forest plot showing the effect of a smoking history on severe COVID‐19. B, Forest plot showing the effect of a smoking history on severe or critical COVID‐19. C, Forest plot showing the effect of a smoking history on mortality. COVID‐19, coronavirus disease 2019

The overall prevalence of a smoking history, including current, former, and/or unspecified smokers, was 26.9% (specifically, China: 10.3%, United States: 23.6%). Their outcomes were investigated in 35 studies. Compared to never‐smokers, a history of smoking significantly increased the risk of presenting with severe disease (RR: 1.31; 95% CI: 1.12‐1.54; *P* = .001; *I* ^2^ = 12%; Figure 3A), as well as severe or critical disease (RR: 1.35; 95% CI: 1.19‐1.53; *P* < .0001; *I* ^2^ = 19%; Figure 3B). However, only the effect on severe or critical disease remained significant when limiting the analysis to only studies using the COVID‐19‐specific criteria for grading severity (Appendix p29). The effect on critical disease alone was not statistically significant (RR: 1.44; 95% CI: 0.95‐2.17; *P* = .085; *I* ^2^ = 0%; Appendix p25). However, a smoking history significantly increased mortality risk (RR: 1.26; 95% CI: 1.20‐1.32; *P* < .0001; *I* ^2^ = 0%; Figure 3C) in addition to other in‐hospital outcomes, such as disease progression (RR: 2.18; 95% CI: 1.06‐4.49; *P* = .035; *I* ^2^ = 69%; Appendix p26) and mechanical ventilation requirement (RR: 1.20; 95% CI: 1.01‐1.42; *P* = .043; *I* ^2^ = 0%; Appendix p28). There was no statistically significant difference in ICU admission (RR: 1.12; 95% CI: 0.96‐1.31; *P* = .157; *I* ^2^ = 0%; Appendix p27). Sensitivity analyses excluding lower‐quality studies supported the primary analyses for all outcomes of interest (Appendix p29). Only the mortality analysis facilitated comparison by country, in which significant detrimental effects were observed in publications from China, United States, and the UK, but not Italy, which contributed one study only for this outcome (Appendix p29).

Overall, there was a moderate‐to‐high degree of heterogeneity between studies evaluating the effects of current smoking and a low degree of heterogeneity between studies investigating a history of smoking. The potential for publication bias was only detected in the comparison of disease progression in patients with a smoking history (Appendix p26), though heterogeneity was high for this outcome.

## 4 DISCUSSION

To our knowledge, this is the largest meta‐analysis amongst peer‐reviewed literature assessing the effect of smoking tobacco on the severity of COVID‐19. Principally, the present analysis found that current smokers have an increased risk of presenting to hospital with severe COVID‐19 and are approximately twice as likely to experience severe or critical COVID‐19 as former or never‐smokers. While this risk became nonsignificant following sensitivity analysis of good‐quality studies only, there were only two studies for each outcome and none graded disease severity by COVID‐19‐specific criteria, thus precluding meaningful interpretation. Overall, there was a high degree of heterogeneity amongst studies evaluating current smoking, even when analyzing good‐quality studies only. For patients with a smoking history, there is an increased risk of presentation to hospital with severe, as well as severe or critical, COVID‐19 and subsequent increased risk of in‐hospital mortality. Additionally, these patients were more likely to experience disease progression and require mechanical ventilation. That all outcomes remained significant on inclusion of only good‐quality studies suggests these analyses represent true effects. A high level of heterogeneity was only observed in assessing the effect of smoking history on disease progression, which is likely secondary to substantial inter‐study variation in defining progression. This outcome also displayed potential for publication bias, however, none was found in other analyses, indicating the low impact of publication bias on our results.

Our finding that current smoking is associated with increased disease severity in COVID‐19 patients validates previous findings from several smaller meta‐analyses in a much larger patient population, achieved through a more rigorous, prospectively registered methodology.^9^, ^10^, ^11^, ^12^, ^21^ The finding that patients with a smoking history are at increased risk of more severe disease, and increased mortality, also confirms previous findings of a smaller meta‐analysis.^11^ The association of both current smoking and smoking history with greater severity of COVID‐19 is biologically plausible for a wealth of reasons. Smoking tobacco is the primary cause of preventable disease, disability, and death in the United States, and is responsible for over 8 million deaths worldwide per year.^68^ Smoking is a major risk factor for adverse respiratory and cardiovascular outcomes, in addition to a wide range of malignant and nonmalignant disease.^68^ In addition, smoking increases severity and mortality of both bacterial and viral infections through the induction of mechanical and structural changes in the respiratory tract and alteration of cell‐ and humoral‐mediated immune responses.^69^, ^70^ In the context of respiratory viruses, smoking has been reported to cause increased hospital and ICU admissions with influenza infection, greater severity with respiratory syncytial virus bronchiolitis and increased mortality with viral pneumonia.^71^, ^72^, ^73^

With regard to coronaviruses, in particular, smoking is associated with increased susceptibility and mortality in MERS‐CoV infection, potentially due to upregulation of dipeptidyl peptidase‐IV, the host receptor for MERS‐CoV, in smokers.^74^, ^75^ The angiotensin‐converting enzyme‐2 (ACE‐2), previously shown to be the host receptor for SARS‐CoV, has also been proven to be the host receptor for SARS‐CoV‐2, facilitating initial intracellular entry via interactions with viral spike glycoproteins.^76^ Subsequent studies have confirmed that ACE‐2 expression is upregulated in human lung tissue samples taken from both current and past smokers, likely mediated by the α‐7 subtype of the nicotinic acetylcholine receptor.^77^, ^78^, ^79^, ^80^, ^81^ In a series of elegant in vitro experiments, Smith et al^80^ report a consistent correlation between smoking history and increased ACE‐2 expression that was dose‐dependent, detectable in both bulk and single‐cell analyses, and remained significant after multivariate linear regression controlling for age, sex, race, and body mass index. It is, therefore, plausible that smokers are exposed to higher SARS‐CoV‐2 loads as a result of increased expression of ACE‐2, which may provide a mechanistic explanation for the increased risk of severe disease and mortality associated with smoking in COVID‐19 patients that we report. Moreover, the inhibition of SARS‐CoV‐2 progression in vitro by human recombinant soluble ACE‐2, a neutralizing agent, holds therapeutic potential and is currently in phase II clinical trials (ClinicalTrials.gov Identifier: NCT04335136).^82^ However, to complicate matters, previous studies also report that postentry viral‐mediated downregulation of ACE‐2 played a major role in the pathogenesis of SARS‐CoV‐associated acute lung injury.^83^, ^84^ Smoking itself has been postulated as having varying, organ‐specific effects on ACE‐2 levels, with specific cigarette components, such as nicotine, potentially exerting a different effect to whole smoke.^80^ Therefore, further studies characterizing the complex relationship of smoking and ACE‐2 in COVID‐19 are warranted.

That smoking history is associated with a significantly increased risk of in‐hospital mortality in COVID‐19 patients, whilst current smoking is not, is a surprising finding. Reductions in morbidity and all‐cause mortality following smoking cessation are well characterized and thus former smokers would be expected to have better baseline health status and improved outcomes.^68^ A systematic review assessing prevalence of current smokers who were hospitalized for COVID‐19 reported a pooled prevalence of 6.5% and propose that in view of the lower than expected prevalence of current smokers compared to population estimates, current smoking is not a predisposing factor for hospitalization and smoking and/or nicotine may exert a protective effect against severe COVID‐19.^85^ The idea that smoking and/or nicotine may be protective against COVID‐19 is echoed by several preprint studies that gained widespread media attention.^14^ Although these hypotheses may explain the nonsignificant association of current smoking and increased mortality that we report, since the majority of included studies did not statistically adjust the effect of smoking for baseline covariates, it is not appropriate to compare the prevalence of smoking in hospitalized COVID‐19 patients with overall population estimates, as the two populations are inherently different with regards to demographic factors. We believe there are far more credible reasons for the nonsignificant association between current smoking and mortality that we report and the low prevalence of smoking among patients with COVID‐19 in published studies.

Predominantly, in the context of a global pandemic, accurately recording smoking history is likely to be of low priority for frontline clinicians whose principal focus is stabilizing severely and critically ill patients. Therefore, patients may have been too acutely unwell to answer questions or clinicians may not enquired directly about smoking status, leading to misclassification of smokers as nonsmokers. Similarly, collateral history collected from family members or referring clinicians is likely to be less accurate than ascertainment of patient‐reported smoking status. Additionally, in an example of reverse causality, hospitalized patients are more likely to have quit smoking on admission, resulting in additional potential misclassification of current smokers as former or nonsmokers. Given the well‐known scarcity of ICU resources such as ventilators, it is also possible that social desirability bias may have contributed to patients not reporting current smoking for fear of being denied access to such interventions, further exacerbating misclassification bias.^14^, ^86^ Finally, given the association of smoking with lower socioeconomic status,^87^ it is possible that current smokers are exhibiting worse health‐seeking behaviors and either self‐treating or deteriorating in the community. Thus, they would not be accounted for in the reported studies which assessed hospitalized patients, leading to survivorship bias and lower event rates for in‐hospital mortality. Due to these factors, the summary estimate for in‐hospital mortality we report has likely been biased towards a null result for current smokers. Similarly, the twofold increase in risk of severe or critical disease for current smokers is likely an underestimate of the effect size.

With no targeted therapies against COVID‐19 currently available, as a scientific community, we must focus on prevention, particularly for those at risk of severe or critical disease. Frontline clinicians must conscientiously record accurate smoking histories in all confirmed COVID‐19 patients, both for triage of vulnerable patients and to support future research efforts. During the current pandemic, independent surveys have reported increased smoking frequency in current smokers and high rates of relapse in former smokers,^88^, ^89^, ^90^ which is unsurprising given the stress, isolation, and other adverse psychosocial repercussions of life during a global pandemic.^91^, ^92^ Considering our finding that current smoking and smoking history are associated with increased COVID‐19 severity, urgent public health measures emphasizing smoking cessation advice, support and pharmacotherapy must be provided to reduce overall disease burden, despite a currently altered social landscape. Good‐quality studies have proven the benefits of mobile phone‐based interventions,^93^ highlighting that even during periods of social distancing and self‐isolation, remote methods of smoking cessation may be feasible and efficacious. Furthermore, as countries begin easing lockdown restrictions, it is imperative that governments and policymakers protect vulnerable populations, such as current and former smokers, through adequate shielding measures and appropriate legislation.

The present analysis has several limitations, principally the use of aggregate data for our meta‐analysis, which precludes adjustment for certain covariates reported to be predictive of disease severity, such as age, gender, and comorbidities,^3^, ^4^, ^5^ and prevents examination of heterogeneity and subgroup analysis at the patient level. The use of individual patient data may have addressed this, however, considering the urgency of our research question and direct applicability to patient care, the considerable time burden associated with conducting an individual patient data meta‐analysis was deemed inappropriate. Also, with most studies reporting on Chinese populations, we cannot exclude the possibility of ethnic differences in smoking and susceptibility to severe COVID‐19 caused by smoking, which may have confounded our analysis. However, this reflects the current landscape of peer‐reviewed literature, which at the present time consists mainly of data from China. We were also unable to assess the effect of e‐cigarettes on COVID‐19 as no studies collected separate data on their usage, which would have been informative considering rises in popularity of these products. Finally, as discussed, the high likelihood of misclassification bias concerning current smoking status across included studies suggests that our analysis potentially underestimates the impact of current smoking on both disease severity and mortality, creating an even more compelling argument for urgent public health measures to support smoking cessation during the present time.

In conclusion, in the largest meta‐analysis available amongst peer‐reviewed literature, we report that both current smoking and a smoking history significantly increased COVID‐19 severity, whilst smoking history also significantly increased mortality risk. Due to problems with potential misclassification of current smokers among included studies, the analysis likely underestimates the likelihood of severity in this patient population. As the COVID‐19 pandemic continues to burden societies worldwide, our analysis suggests that smoking represents one of the most immediately modifiable risk factors to reduce the substantial morbidity associated with the disease. In light of this finding, governments, policymakers, and other key stakeholders must ensure that appropriate measures are taken to support and maintain smoking cessation to protect vulnerable populations and reduce the strain placed on healthcare systems working at full capacity during this global crisis.

## CONFLICT OF INTERESTS

The authors declare that there are no conflict of interests.

## AUTHOR CONTRIBUTIONS

AK conceptualized the work. RKR, WNC, AS, AD, PTS, and AK were responsible for acquisition, analysis, and interpretation of data. RKR, WNC, and AK drafted the manuscript. AS, AD, PTS, and AK provided critical revisions. All authors gave final approval of the version to be published and agree to be accountable for all aspects of the work.

## Supporting information

Supporting information

## Data Availability

The data that support the findings of this study are available from the corresponding author upon reasonable request.
