# Supplementary material for: The effect of smoking on COVID‐19 severity: A systematic review and meta‐analysis
Source: J Med Virol. 2020 Aug 13;93(2):1045–56. doi: 10.1002/jmv.26389 (PMC7436545; doi:10.1002/jmv.26389)
Supplement: Supplementary file 1 — Supporting information [file JMV-93-1045-s001.pdf]

SUPPLEMENTARY APPENDIX

The effect of smoking on COVID-19 severity: a systematic review and meta-analysis

Rohin K. Reddy*, Walton N. Charles*, Alexandros Sklavounos, Atul Dutt, Paul T. Seed and Ankur Khajuria
*Rohin K. Reddy and Walton N. Charles are co-first authors and contributed equally to this work.

Table of Contents

Page

1. **Summary of previous systematic reviews**
   i. Table S1: Key findings of previous reviews 2
   ii. Table S2: Quality of reviews according to AMSTAR 2 criteria 3

2. **Supplementary methods**
   i. PROSPERO review protocol 4
   ii. Table S3: PRISMA checklist 8
   iii. AMSTAR 2 checklist 9
   iv. Search strategy for MEDLINE and Embase 13
   v. Diagnostic criteria for grading disease severity 13
   vi. Table S4: Study definitions of disease progression 14
   vii. Table S5: Modified Newcastle-Ottawa Scale for case series 14
   viii. Table S6: Modified Newcastle-Ottawa Scale for cohort studies 15
   ix. Table S7: Modified Newcastle-Ottawa Scale for cross-sectional studies 15
   x. Conversion of Newcastle-Ottawa Scale scores to overall grade 16

3. **Supplementary results**
   i. Table S8: Breakdown of Newcastle-Ottawa Scale grading for included studies 17
   ii. Figure S1: Funnel plots for the effect of current smoking on
      A. Severe COVID-19 18
      B. Severe or critical COVID-19 18
      C. Mortality 18
   iii. Figure S2: Forest and funnel plots for the effect of current smoking on
      A. Disease progression 19
      B. ICU admission 20
      C. Mechanical ventilation requirement 21
   iv. Table S9: Sensitivity analyses of the effect of current smoking 22
   v. Figure S3: Forest plot showing the effect of current smoking on mortality by country 23
   vi. Figure S4: Funnel plots for the effect of a smoking history on
      A. Severe COVID-19 24
      B. Severe or critical COVID-19 24
      C. Mortality 24
   vii. Figure S5: Forest and funnel plots for the effect of a smoking history on
      A. Critical COVID-19 alone 25
      B. Disease progression 26
      C. ICU admission 27
      D. Mechanical ventilation requirement 28
   viii. Table S10: Sensitivity analyses of the effect of a smoking history 29
   ix. Figure S6: Forest plot showing the effect of a smoking history on mortality by country 29

4. **Supplementary references** 30

SUMMARY OF PREVIOUS SYSTEMATIC REVIEWS

Table S1: Key findings of previous reviews assessing the effect of smoking on COVID-19 severity

	Databases and dates of search	Inclusion criteria	Studies included (patients included)	Outcomes studied	Key findings
**Karanasos et al.**[1]	PubMed, Cochrane and handsearch of included studies' reference lists from Sept 1, 2019 to May 4, 2020	English language studies reporting on hospitalized patients with COVID-19 that reported smoking status stratified by component of composite outcome or mortality	22 (7146)	1. Composite endpoint for severity (severe and critical cases, ICU admission or invasive ventilation and adverse disease progression or refractory disease)<br>2. Mortality	▪ Composite endpoint for smoking history – modestly increased risk (OR 1.34, 95% CI 1.07-1.67)<br>▪ Composite endpoint for current smoking – non-significant<br>▪ Mortality for smoking history – non-significant<br>▪ Mortality for current smoking – non-significant
**Patanavanich and Glantz**[2]	PubMed between Jan 1, 2020 and Apr 28, 2020	Inclusion criteria not well-defined. Included papers with data on smoking behaviour and COVID-19 disease progression. 17 studies on hospitalized patients, 2 on hospitalized patients and outpatients	19 (11590)	Progression of COVID-19 to more severe or critical conditions or death	▪ Progression of COVID-19 for smoking history – increased risk (OR 1.91, 95% CI 1.42-2.59)<br>▪ Progression of COVID-19 for current smoking – increased risk (OR 1.91, 95% CI 1.10-3.29)
**Zheng et al.**[3]	PubMed, Embase, Web of Science, and CNKI between Jan 1, 2020 and Mar 20, 2020	Studies reporting on COVID-19 patients grouped into critical illness or death and non-critical illness with at least one outcome reported among demographical characteristics, comorbidities, clinical manifestations or laboratory examinations	13 (3027) overall and 5 (1712) that reported data on current smoking	Not well defined. Aimed to identify a range of clinical and laboratory risk factors for COVID-19 patients to develop critical disease or death	Critical illness and mortality for current smoking – increased risk (OR 2.04, 95% CI 1.32-3.15)
**Zhao et al.**[4]	PubMed, Web of Science, Cochrane, WanFang Database, and CNKI between Dec 2019 and Mar 22, 2020	Studies presenting data on COPD or smoking history in COVID-19 cases with or without severe presentation	11 (2002) overall and 7 (1726) that reported data on current smoking	Development of severe COVID-19 as diagnosed according to the guidance issued by the Chinese National Health Committee along with either the requirement of intensive care/ mechanical ventilation or resulting in death	▪ Severe COVID-19 for current smoking – increased risk (OR 1.98, 95% CI 1.29-3.05)<br>▪ Severe COVID-19 for current smoking on sensitivity analysis after excluding the largest study – non-significant
**Lippi and Henry**[5]	PubMed, Scopus, Web of Science and handsearch of included studies' reference lists between 2019 and Mar 9, 2020	Studies reporting the rate of active smokers in COVID-19 patients with clinically validated definition of severe disease	5 (1399)	Not well defined. Aimed to identify effect of smoking on likelihood of developing severe COVID-19 according to a clinically validated definition of severe disease	Severe COVID-19 for current smoking – non-significant
**Vardavas and Nikitara**[6]	PubMed, ScienceDirect and handsearch of included studies' reference lists on Mar 17, 2020	English-language studies published in 2019 and 2020 that included information on patients' smoking status	5 (1549)	Severity of COVID-19, need for mechanical ventilation, need for intensive care unit hospitalization, and death	Did not perform a formal meta-analysis but conclude that smoking is most likely associated with the negative progression and adverse outcomes of COVID-19

Table S2: **Quality of reviews, including the present one, according to AMSTAR 2 criteria[7]**

AMSTAR 2 criterion	Our review	Vardavas and Nikitara[6]	Lippi and Henry[5]	Zhao et al.[4]	Zheng et al.[3]	Patanavanich and Glantz[2]	Karanasos et al.[1]
**1. Did the research questions and inclusion criteria for the review include the components of PICO?**	Yes	Yes	Yes	Yes	Yes	Yes	Yes
**2. Did the report of the review contain an explicit statement that the review methods were established prior to the conduct of the review and did the report justify any significant deviations from the protocol?**	Yes	No	No	No	No	No	No
**3. Did the review authors explain their selection of the study designs for inclusion in the review?**	Yes	Yes	Yes	Yes	Yes	Yes	Yes
**4. Did the review authors use a comprehensive literature search strategy?**	Partial Yes	Partial Yes	Partial Yes	Partial Yes	Partial Yes	No	Partial Yes
**5. Did the review authors perform study selection in duplicate?**	Yes	No	No	Yes	No	No	Yes
**6. Did the review authors perform data extraction in duplicate?**	Yes	No	No	Yes	Yes	No	Yes
**7. Did the review authors provide a list of excluded studies and justify the exclusions?**	No	No	No	No	No	No	No
**8. Did the review authors describe the included studies in adequate detail?**	Yes	Yes	No	Yes	Yes	Yes	Yes
**9. Did the review authors use a satisfactory technique for assessing the risk of bias (RoB) in individual studies that were included in the review?**	Yes	No	No	No	No	No	Yes
**10. Did the review authors report on the sources of funding for the studies included in the review?**	Yes	No	No	No	No	No	No
**11. If meta-analysis was performed, did the review authors use appropriate methods for statistical combination of results?**	Yes	N/A	Yes	Yes	Yes	Yes	Yes
**12. If meta-analysis was performed, did the review authors assess the potential impact of RoB in individual studies on the results of the meta-analysis or other evidence synthesis?**	Yes	N/A	No	No	No	No	No
**13. Did the review authors account for RoB in individual studies when interpreting/discussing the results of the review?**	Yes	No	No	No	Yes	No	Yes
**14. Did the review authors provide a satisfactory explanation for, and discussion of, any heterogeneity observed in the results of the review?**	Yes	N/A	Yes	Yes	Yes	Yes	Yes
**15. If they performed quantitative synthesis, did the review authors carry out an adequate investigation of publication bias (small study bias) and discuss its likely impact on the results of the review?**	Yes	N/A	No	Yes	No	Yes	Yes
**16. Did the review authors report any potential sources of conflict of interest, including any funding they received for conducting the review?**	Yes	Yes	Yes	Yes	No	Yes	Yes
**Overall quality**	**High**	**Critically low**	**Critically low**	**Critically low**	**Low**	**Critically low**	**Moderate**

Criteria for grading overall quality:

- High – *No or one non-critical weakness:* The systematic review provides an accurate and comprehensive summary of the results of the available studies that address the question of interest.
- Moderate – *More than one non-critical weakness**:* The systematic review has more than one weakness, but no critical flaws. It may provide an accurate summary of the results of the available studies that were included in the review.
- Low – *One critical flaw with or without non-critical weaknesses:* The review has a critical flaw and may not provide an accurate and comprehensive summary of the available studies that address the question of interest.
- Critically low – *More than one critical flaw with or without non-critical weaknesses:* The review has more than one critical flaw and should not be relied on to provide an accurate and comprehensive summary of the available studies.
- *Note: Multiple non-critical weaknesses may diminish confidence in the review and it may be appropriate to move the overall appraisal down from moderate to low confidence.

SUPPLEMENTARY METHODS

PROSPERO review protocol (CRD42020180920)[8]

The effects of smoking on COVID-19 severity: a systematic review and meta-analysis

Ankur Khajuria, Walton Charles, Alexandros Sklavounos, Rohin Reddy

Citation

Ankur Khajuria, Walton Charles, Alexandros Sklavounos, Rohin Reddy. The effects of smoking on COVID-19 severity: a systematic review and meta-analysis. PROSPERO 2020 CRD42020180920 Available from: https://www.crd.york.ac.uk/prospero/display_record.php?ID=CRD42020180920

Review question

How does a social history of smoking in patients diagnosed with COVID-19 affect disease severity?

This review will include studies comparing COVID-19 patients who have an active smoking status to those who are non-smokers.

The main outcome will be disease severity.

Searches

Electronic databases will be searched including: MEDLINE, Embase, the Cochrane Central Register of Controlled Trials (CENTRAL) and Web of Science.

This will be undertaken in April.

Comprehensive search terms for “smoking” and “COVID-19” will be utilised.

The search will be restricted to papers available in English, and published from December 2019 onwards.

Types of study to be included

Inclusion criteria: clinical studies (randomised controlled trials, prospective and retrospective comparative observational studies, case series) reporting the smoking status of patients with different severities of COVID-19.

Exclusion criteria: review articles, conference abstracts, opinion pieces, non-clinical studies, studies that do not report on outcomes or smoking status, studies examining other coronaviruses (SARS/MERS), and studies in other languages with no English translations.

Condition or domain being studied

COVID-19 is an acute respiratory disease that has a high infection rate.

The first clinical cases were reported in Wuhan, China, at the end of 2019 and since then it has spread worldwide causing a pandemic.

Common clinical features include cough and dyspnoea, fever, myalgia, sore throat, anosmia and headache.

Most common complications include viral pneumonia, which can evolve into acute respiratory distress syndrome, septic shock, and multi-organ failure.

Worse outcomes are more common in people aged over 65 or in those with co-morbidities, although the

effect of smoking status is currently unclear.

Participants/population
COVID-19 patients who are smokers.

Intervention(s), exposure(s)
Active smoking in COVID-19 patients.

Comparator(s)/control
COVID-19 patients who are non-smokers.

Main outcome(s)
Disease severity, as based on the Chinese Clinical Guidance for COVID-19 Pneumonia Diagnosis and Treatment (Chinese National Health Commission).

Other well-established criteria include: BTS CURB65 score, IDSA/ATS criteria and Pneumonia Severity Index.

In the absence of well-established criteria, clinical endpoints of ICU admission, requirements for mechanical ventilation and/or death will be used as surrogate markers for severity.

* Measures of effect
Odds ratios.

Additional outcome(s)
None.

* Measures of effect
Not applicable.

Data extraction (selection and coding)
Two reviewers will independently screen the titles and abstracts of the papers retrieved in the searches for eligibility.

The full-texts of papers identified as being potentially eligible for inclusion will then be screened for inclusion. Any disagreements between the two authors will be resolved by discussion with a third reviewer.

Data will then be extracted from the studies selected for inclusion, as follows: first author, year of publication, study design, ethics approval, study setting, study population including multicentre studies, participant demographics (including smoking status), disease severity and clinical outcomes (ICU admission, mechanical ventilation requirement, and death).

Risk of bias (quality) assessment
The GRADE (Grading of Recommendations, Assessment, Development and Evaluations) framework will be used to evaluate the quality of each study based on: risk of bias, imprecision, inconsistency, indirectness and publication bias.

Studies will be given a level of evidence of very low, low, moderate or high.

Strategy for data synthesis
COVID-19 outcomes will be compared between smokers and non-smokers.

Outcome data will be pooled using Review Manager software (The Cochrane Collaboration, Denmark) and evaluated using odds ratios with 95% confidence intervals.

Heterogeneity between studies will be assessed in Review Manager, with a meta-analysis performed if the studies are relatively homogenous regarding methodology and outcomes.

The results of the meta-analyses will be shown as forest plots.

If, however, a meta-analysis should prove to be inappropriate, a narrative synthesis will be performed instead.

Analysis of subgroups or subsets
None planned.

Contact details for further information
Alexandros Sklavounos
sklavounosalexandros@gmail.com

Organisational affiliation of the review
Kings College London
https://www.kcl.ac.uk/

Review team members and their organisational affiliations
Dr Ankur Khajuria. Kellogg College, University of Oxford
Dr Walton Charles. Imperial College London
Dr Alexandros Sklavounos. Kings College London
Mr Rohin Reddy. Hull York Medical School, University of York

Type and method of review
Epidemiologic, Meta-analysis, Systematic review

Anticipated or actual start date
04 April 2020

Anticipated completion date
24 May 2020

Funding sources/sponsors
None

Conflicts of interest
Not Applicable
None known

Language
English

Country
England

Stage of review
Review Ongoing

Subject index terms status
Subject indexing assigned by CRD

Subject index terms
Cigarette Smoking; Coronavirus; Coronavirus Infections; Disease Progression; Humans; Public Health; Risk; Risk Factors; Smoking; Tobacco Smoking

Date of registration in PROSPERO
27 April 2020

Date of first submission
24 April 2020

Stage of review at time of this submission

Stage	Started	Completed
Preliminary searches	Yes	No
Piloting of the study selection process	No	No
Formal screening of search results against eligibility criteria	No	No
Data extraction	No	No
Risk of bias (quality) assessment	No	No
Data analysis	No	No

The record owner confirms that the information they have supplied for this submission is accurate and complete and they understand that deliberate provision of inaccurate information or omission of data may be construed as scientific misconduct.

The record owner confirms that they will update the status of the review when it is completed and will add publication details in due course.

Versions

27 April 2020

PROSPERO
This information has been provided by the named contact for this review. CRD has accepted this information in good faith and registered the review in PROSPERO. The registrant confirms that the information supplied for this submission is accurate and complete. CRD bears no responsibility or liability for the content of this registration record, any associated files or external websites.

NB: Since the protocol was registered, a significant number of papers were found to report patient outcomes by smoking history. Hence, in addition to analysing the effect of current smoking, a separate analysis was conducted comparing those with a smoking history (current and former smokers) with never-smokers.

Table S3: **PRISMA checklist**[9]

PRISMA section	Checklist item	Reported on page no.
**Title**		
1. Title	Identify the report as a systematic review, meta-analysis, or both.	1
**Abstract**		
2. Structured summary	Provide a structured summary including, as applicable: background; objectives; data sources; study eligibility criteria, participants, and interventions; study appraisal and synthesis methods; results; limitations; conclusions and implications of key findings; systematic review registration number.	4-5
**Introduction**		
3. Rationale	Describe the rationale for the review in the context of what is already known.	6
4. Objectives	Provide an explicit statement of questions being addressed with reference to participants, interventions, comparisons, outcomes, and study design (PICOS).	7
**Methods**		
5. Protocol and registration	Indicate if a review protocol exists, if and where it can be accessed (e.g., Web address), and, if available, provide registration information including registration number.	10<br>Supp. (pp4-7)
6. Eligibility criteria	Specify study characteristics (e.g., PICOS, length of follow-up) and report characteristics (e.g., years considered, language, publication status) used as criteria for eligibility, giving rationale.	8
7. Information sources	Describe all information sources (e.g., databases with dates of coverage, contact with study authors to identify additional studies) in the search and date last searched.	8
8. Search	Present full electronic search strategy for at least one database, including any limits used, such that it could be repeated.	Supp. (p13)
9. Study selection	State the process for selecting studies (i.e., screening, eligibility, included in systematic review, and, if applicable, included in the meta-analysis).	9
10. Data collection process	Describe method of data extraction from reports (e.g., piloted forms, independently, in duplicate) and any processes for obtaining and confirming data from investigators.	9
11. Data items	List and define all variables for which data were sought (e.g., PICOS, funding sources) and any assumptions and simplifications made.	9
12. Risk of bias in individual studies	Describe methods used for assessing risk of bias of individual studies (including specification of whether this was done at the study or outcome level), and how this information is to be used in any data synthesis.	9
13. Summary measures	State the principal summary measures (e.g., risk ratio, difference in means).	10
14. Synthesis of results	Describe the methods of handling data and combining results of studies, if done, including measures of consistency (e.g., I^2) for each meta-analysis.	10
15. Risk of bias across studies	Specify any assessment of risk of bias that may affect the cumulative evidence (e.g., publication bias, selective reporting within studies).	10
16. Additional analyses	Describe methods of additional analyses (e.g., sensitivity or subgroup analyses, meta-regression), if done, indicating which were pre-specified.	10
**Results**		
17. Study selection	Give numbers of studies screened, assessed for eligibility, and included in the review, with reasons for exclusions at each stage, ideally with a flow diagram.	11<br>Figure 1
18. Study characteristics	For each study, present characteristics for which data were extracted (e.g., study size, PICOS, follow-up period) and provide the citations.	Table 1
19. Risk of bias within studies	Present data on risk of bias of each study and, if available, any outcome level assessment (see item 12).	Table 1<br>Supp. (p17)
20. Results of individual studies	For all outcomes considered (benefits or harms), present, for each study: (a) simple summary data for each intervention group (b) effect estimates and confidence intervals, ideally with a forest plot.	Figures 2&3<br>Supp. (figures S2&3, S5&6)
21. Synthesis of results	Present results of each meta-analysis done, including confidence intervals and measures of consistency.	12-13
22. Risk of bias across studies	Present results of any assessment of risk of bias across studies (see Item 15).	13
23. Additional analysis	Give results of additional analyses, if done (e.g., sensitivity or subgroup analyses, meta-regression [see Item 16]).	12-13
**Discussion**		
24. Summary of evidence	Summarize the main findings including the strength of evidence for each main outcome; consider their relevance to key groups (e.g., healthcare providers, users, and policy makers).	14
25. Limitations	Discuss limitations at study and outcome level (e.g., risk of bias), and at review-level (e.g., incomplete retrieval of identified research, reporting bias).	18-19
26. Conclusions	Provide a general interpretation of the results in the context of other evidence, and implications for future research.	19
**Funding**		
27. Funding	Describe sources of funding for the systematic review and other support (e.g., supply of data); role of funders for the systematic review.	20

AMSTAR 2 checklist[7]

You are viewing as a guest Login

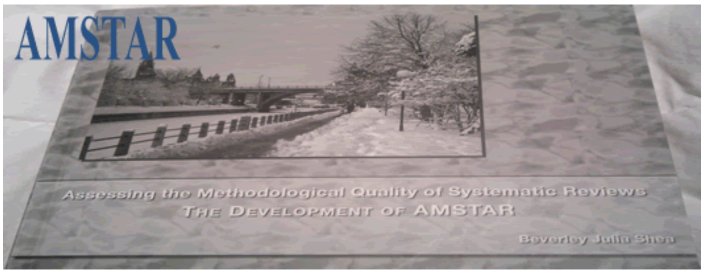

Home | About Us | Publications | Checklist | FAQs | Contact Us

AMSTAR Checklist

Printer Friendly Version

Article Name: The effect of smoking on COVID-19 severity

---

1. Did the research questions and inclusion criteria for the review include the components of PICO?

For Yes:
- [x] Population
- [x] Intervention
- [x] Comparator group
- [x] Outcome

Optional (recommended)
- [ ] Timeframe for follow up

- [x] Yes
- [ ] No

---

2. Did the report of the review contain an explicit statement that the review methods were established prior to the conduct of the review and did the report justify any significant deviations from the protocol?

For Partial Yes:
The authors state that they had a written protocol or guide that included ALL the following:
- [x] review question(s)
- [x] a search strategy
- [x] inclusion/exclusion criteria
- [x] a risk of bias assessment

For Yes:
As for partial yes, plus the protocol should be registered and should also have specified:
- [x] a meta-analysis/synthesis plan, if appropriate, and
- [x] a plan for investigating causes of heterogeneity
- [x] a plan for investigating causes of heterogeneity

- [x] Yes
- [ ] Partial Yes
- [ ] No

---

3. Did the review authors explain their selection of the study designs for inclusion in the review?

For Yes, the review should satisfy ONE of the following:
- [ ] Explanation for including only RCTs
- [ ] OR Explanation for including only NRSI
- [x] OR Explanation for including both RCTs and NRSI

- [x] Yes
- [ ] No

---

4. Did the review authors use a comprehensive literature search strategy?

For Partial Yes (all the following):
- [x] searched at least 2 databases (relevant to research question)
- [x] provided key word and/or search strategy

For Yes, should also have (all the following):
- [x] searched the reference lists / bibliographies of included studies
- [x] searched trial/study registries

- [ ] Yes
- [x] Partial Yes
- [ ] No

☑ justified publication restrictions (e.g. language)	☐ included/consulted content experts in the field<br>☐ where relevant, searched for grey literature<br>☑ conducted search within 24 months of completion of the review	

5. Did the review authors perform study selection in duplicate?

For Yes, either ONE of the following:

☑ at least two reviewers independently agreed on selection of eligible studies and achieved consensus on which studies to include<br>☐ OR two reviewers selected a sample of eligible studies and achieved good agreement (at least 80 percent), with the remainder selected by one reviewer.	☑ Yes<br>☐ No

6. Did the review authors perform data extraction in duplicate?

For Yes, either ONE of the following:

☑ at least two reviewers achieved consensus on which data to extract from included studies<br>☐ OR two reviewers extracted data from a sample of eligible studies and achieved good agreement (at least 80 percent), with the remainder extracted by one reviewer.	☑ Yes<br>☐ No

7. Did the review authors provide a list of excluded studies and justify the exclusions?

For Partial Yes:	For Yes, must also have:	
☐ provided a list of all potentially relevant studies that were read in full-text form but excluded from the review	☐ Justified the exclusion from the review of each potentially relevant study	☐ Yes<br>☐ Partial Yes<br>☑ No

8. Did the review authors describe the included studies in adequate detail?

For Partial Yes (ALL the following):	For Yes, should also have ALL the following:	
☑ described populations	☑ described population in detail	☑ Yes<br>☐ Partial Yes<br>☐ No
☑ described interventions	☑ described intervention in detail (including doses where relevant)	
☑ described comparators	☑ described comparator in detail (including doses where relevant)	
☑ described outcomes	☑ described study's setting	
☑ described research designs	☑ timeframe for follow-up	

9. Did the review authors use a satisfactory technique for assessing the risk of bias (RoB) in individual studies that were included in the review?

RCTs

For Partial Yes, must have assessed RoB from	For Yes, must also have assessed RoB from:	
☐ unconcealed allocation, and	☐ allocation sequence that was not truly random, and	☐ Yes<br>☐ Partial Yes<br>☐ No<br>☑ Includes only NRSI
☐ lack of blinding of patients and assessors when assessing outcomes (unnecessary for objective outcomes such as all-cause mortality)	☐ selection of the reported result from among multiple measurements or analyses of a specified outcome	

NRSI

For Partial Yes, must have assessed RoB:	For Yes, must also have assessed RoB:	
☑ from confounding, and	☑ methods used to ascertain exposures and outcomes, and	☑ Yes<br>☐ Partial Yes<br>☐ No
☑ from selection bias	☑ selection of the reported result from among multiple measurements or analyses	

of a specified outcome	☐ Includes only RCTs

10. Did the review authors report on the sources of funding for the studies included in the review?

For Yes

☑ Must have reported on the sources of funding for individual studies included in the review. Note: Reporting that the reviewers looked for this information but it was not reported by study authors also qualifies	☑ Yes<br>☐ No

11. If meta-analysis was performed did the review authors use appropriate methods for statistical combination of results?

RCTs

For Yes:

☐ The authors justified combining the data in a meta-analysis<br>☐ AND they used an appropriate weighted technique to combine study results and adjusted for heterogeneity if present.<br>☐ AND investigated the causes of any heterogeneity	☐ Yes<br>☐ No<br>☐ No meta-analysis conducted

For NRSI

For Yes:

☑ The authors justified combining the data in a meta-analysis<br>☑ AND they used an appropriate weighted technique to combine study results, adjusting for heterogeneity if present<br>☑ AND they statistically combined effect estimates from NRSI that were adjusted for confounding, rather than combining raw data, or justified combining raw data when adjusted effect estimates were not available<br>☑ AND they reported separate summary estimates for RCTs and NRSI separately when both were included in the review	☑ Yes<br>☐ No<br>☐ No meta-analysis conducted

12. If meta-analysis was performed, did the review authors assess the potential impact of RoB in individual studies on the results of the meta-analysis or other evidence synthesis?

For Yes:

☐ included only low risk of bias RCTs<br>☑ OR, if the pooled estimate was based on RCTs and/or NRSI at variable RoB, the authors performed analyses to investigate possible impact of RoB on summary estimates of effect.	☑ Yes<br>☐ No<br>☐ No meta-analysis conducted

13. Did the review authors account for RoB in individual studies when interpreting/ discussing the results of the review?

For Yes:

☐ included only low risk of bias RCTs<br>☑ OR, if RCTs with moderate or high RoB, or NRSI were included the review provided a discussion of the likely impact of RoB on the results	☑ Yes<br>☐ No

14. Did the review authors provide a satisfactory explanation for, and discussion of, any heterogeneity observed in the results of the review?

For Yes:

☐ There was no significant heterogeneity in the results<br>☑ OR if heterogeneity was present the authors performed an investigation of sources of any heterogeneity in the results and discussed the impact of this on the results of the review	☑ Yes<br>☐ No

15. If they performed quantitative synthesis did the review authors carry out an adequate investigation of publication bias (small study bias) and discuss its likely impact on the results of the review?

For Yes:

- [x] performed graphical or statistical tests for publication bias and discussed the likelihood and magnitude of impact of publication bias

- [x] Yes
- [ ] No
- [ ] No meta-analysis conducted

---

16. Did the review authors report any potential sources of conflict of interest, including any funding they received for conducting the review?

For Yes:

- [x] The authors reported no competing interests OR
- [ ] The authors described their funding sources and how they managed potential conflicts of interest

- [x] Yes
- [ ] No

To cite this tool: Shea BJ, Reeves BC, Wells G, Thuku M, Hamel C, Moran J, Moher D, Tugwell P, Welch V, Kristjansson E, Henry DA. AMSTAR 2: a critical appraisal tool for systematic reviews that include randomised or non-randomised studies of healthcare interventions, or both. BMJ. 2017 Sep 21;358:j4008.

Calculate

Copyright © 2017 AMSTAR All Rights Reserved |

Search strategy for MEDLINE and Embase

1. smoker.mp. [mp=ti, ab, hw, tn, ot, dm, mf, dv, kw, fx, dq, nm, kf, ox, px, rx, an, ui, sy]
2. smoking.mp. [mp=ti, ab, hw, tn, ot, dm, mf, dv, kw, fx, dq, nm, kf, ox, px, rx, an, ui, sy]
3. tobacco.mp. [mp=ti, ab, hw, tn, ot, dm, mf, dv, kw, fx, dq, nm, kf, ox, px, rx, an, ui, sy]
4. cigarette.mp. [mp=ti, ab, hw, tn, ot, dm, mf, dv, kw, fx, dq, nm, kf, ox, px, rx, an, ui, sy]
5. risk factors.mp. [mp=ti, ab, hw, tn, ot, dm, mf, dv, kw, fx, dq, nm, kf, ox, px, rx, an, ui, sy]
6. coronavirus.mp. [mp=ti, ab, hw, tn, ot, dm, mf, dv, kw, fx, dq, nm, kf, ox, px, rx, an, ui, sy]
7. coronavirus*.mp. [mp=ti, ab, hw, tn, ot, dm, mf, dv, kw, fx, dq, nm, kf, ox, px, rx, an, ui, sy]
8. COVID-19.mp. [mp=ti, ab, hw, tn, ot, dm, mf, dv, kw, fx, dq, nm, kf, ox, px, rx, an, ui, sy]
9. 2019-nCoV.mp. [mp=ti, ab, hw, tn, ot, dm, mf, dv, kw, fx, dq, nm, kf, ox, px, rx, an, ui, sy]
10. SARS-CoV-2.mp. [mp=ti, ab, hw, tn, ot, dm, mf, dv, kw, fx, dq, nm, kf, ox, px, rx, an, ui, sy]
11. Coronavirus Infections.mp. [mp=ti, ab, hw, tn, ot, dm, mf, dv, kw, fx, dq, nm, kf, ox, px, rx, an, ui, sy]

12. 1 or 2 or 3 or 4 or 5
13. 6 or 7 or 8 or 9 or 10 or 11
14. 12 and 13

Diagnostic criteria for grading disease severity

Chinese COVID-19-specific criteria[10]

- Severe disease defined by ANY of the following factors:
    1. Dyspnoea, with a respiratory rate ≥ 30 breaths/min
    2. Oxygen saturation ≤ 93% at rest
    3. Ratio of partial pressure of arterial oxygen to the fraction of inspired oxygen (PaO_2/FiO_2 ratio) ≤ 300
- Critical disease defined by ANY of the following factors:
    1. Respiratory failure necessitating mechanical ventilation
    2. Shock
    3. ICU monitoring and treatment, in combination with other organ failure

Infectious Diseases Society of America/American Thoracic Society (IDSA/ATS) criteria for severe community-acquired pneumonia[11]

- Severe disease defined as one major OR three or more minor criteria
- Major criteria:
    1. Respiratory failure necessitating mechanical ventilation
    2. Septic shock with need for vasopressors
- Minor criteria:
    1. Respiratory rate ≥ 30 breaths/min
    2. PaO_2/FiO_2 ratio ≤ 250
    3. Multilobar infiltrates
    4. Confusion/disorientation
    5. Uraemia (blood urea nitrogen level ≥ 20 mg/dl)
    6. Leukopenia due to infection alone (white blood cell count < 4000 cells/μl)
    7. Thrombocytopenia (platelet count < 100000/μl)
    8. Hypothermia (core temperature < 36°C)
    9. Hypotension requiring aggressive fluid resuscitation

Of all included studies[12-58], only Toussie et al.[44] used a locally devised criteria based on chest X-ray findings

- Each lung was divided into three zones (upper zone from superior hilar markings to the apices, middle zone from inferior to superior hilar markings and lower zone from the costophrenic sulcus to inferior hilar markings), with each zone graded on whether an opacity was absent (0) or present (1)
- Total scores were calculated (maximum of 6)
- Severe cases defined as a score of 3-6

Due to variations in the diagnostic criteria for grading severity (e.g. any patient meeting the major criteria of the IDSA/ATS criteria for 'severe' disease would actually be classified as having 'critical' disease according to the Chinese COVID-19-specific criteria), we performed further sensitivity analyses restricting to only studies using the Chinese criteria developed specifically to evaluate COVID-19 severity.

Table S4: Study definitions of disease progression

	Definition
**Bi X et al.[14]**	▪ Progression from non-severe to severe or critical disease<br>▪ Severity graded according to the Chinese COVID-19-specific criteria
**Huang J et al.[27]**	▪ Required oxygen support (including high-flow oxygen supply, as well as invasive or non-invasive mechanical ventilation)
**Ji D et al.[31]**	▪ Progression from non-severe to severe disease<br>▪ Severity graded according to the Chinese COVID-19-specific criteria, with additional criteria including mechanical ventilation requirement or worsening lung CT scan findings
**Liu W et al.[37]**	▪ Progression from non-severe to severe or critical disease or death<br>▪ Progression from severe to critical disease or death<br>▪ Progression from critical disease to death<br>▪ Severity graded according to the Chinese COVID-19-specific criteria
**Yu T et al.[51]**	▪ Worsening lung CT scan findings 1-week post-treatment
**Yu X et al.[52]**	▪ Progression from non-severe (mild/moderate) to severe disease<br>▪ Severity criteria not specified
**Zheng Y et al.[57]**	▪ Progression from non-severe to severe or critical disease<br>▪ Severity graded according to the Chinese COVID-19-specific criteria

Table S5: Modified Newcastle-Ottawa Scale for case series[59]

	**Scoring components (any to score)**	**Non-scoring components**
**Selection (out of 4)**		
1. Is the case definition adequate?	▪ Independent validation (e.g. more than 1 person/record/time/process used to extract information)<br>▪ Reference to primary record source	▪ Record linkage (e.g. ICD codes in databases of unverifiable registry-based studies)<br>▪ Self-report with no reference to primary record<br>▪ No description
2. Representativeness of the cases	▪ Consecutive/obviously representative series<br>▪ All eligible cases with outcome of interest over defined period of time/hospital	▪ Only investigated a specific population of hospitalised COVID-19 patients (only those with e.g. cancer/IBD/admitted to ICU etc.)<br>▪ Potential for selection bias or not stated
3. Selection of controls	▪ Community controls (i.e. same community as cases and would be cases if they had outcome)	▪ Hospital controls<br>▪ No description
4. Definition of controls	▪ No history of study endpoint stated	▪ No description
**Comparability (out of 2)**		
5. Age	▪ Adjusted for age in multivariate model<br>▪ Exposed and unexposed cases matched in design by age	▪ Statements of no difference between groups/ differences were not statistically significant
6. Any other significant risk factor	▪ As above, but for any other significant risk factor (such as gender or comorbidities)	▪ Statements of no difference between groups/ differences were not statistically significant
**Exposure (out of 3)**		
7. Ascertainment of exposure	▪ Secure record<br>▪ Structured interview conducted blind to case/ control status	▪ Self-reported<br>▪ Interview not blinded to case/control<br>▪ No description
8. Same method of ascertainment for cases and controls	▪ Yes	▪ Different method used
9. Non-response rate	▪ Same rates for both groups	▪ Different rates, even if difference explained

Table S6: **Modified Newcastle-Ottawa Scale for cohort studies**[59]

	Scoring components (any to score)	Non-scoring components
**Selection (out of 4)**		
1. Representativeness of the exposed cohort	▪ Representative cohort of COVID-19 patients	▪ Specific cohort of hospitalised COVID-19 patients (only those with e.g. cancer/IBD/ admitted to ICU etc.)<br>▪ No description
2. Selection of the non-exposed cohort	▪ Drawn from the same community as the above cohort	▪ Drawn from a different community (e.g. compared patients to healthcare staff)<br>▪ No description
3. Ascertainment of exposure	▪ Secure record<br>▪ Structured interview	▪ Written self-report<br>▪ No description
4. Demonstration that outcome of interest was not present at start of study	▪ Stated (e.g. in studies of progression to severe disease, must state that only mild/moderate disease patients were included or that severe disease patients were not included)	▪ Not stated
**Comparability (out of 2)**		
5. Age	▪ Adjusted for age in multivariate model<br>▪ Cohorts matched by age in design	▪ Statements of no difference between groups/ differences were not statistically significant
6. Any other significant risk factor	▪ As above, but for any other significant risk factor (such as gender or comorbidities)	▪ Statements of no difference between groups/ differences were not statistically significant
**Outcome (out of 3)**		
7. Assessment of outcome	▪ Secure record/database<br>▪ Independent assessment (e.g. more than 1 person/record/time/process)	▪ Self-reported<br>▪ No reference to original medical records<br>▪ No description of how outcomes extracted
8. Was follow-up long enough for outcomes to occur?	▪ Follow-up of at least 30 days stated/calculated from last date of enrolment<br>NB: Short follow-up selected in recognition of current urgency of COVID-19 research	▪ Follow-up less than 30 days<br>▪ Patients still in hospital at the end of the study period with potential for outcomes of interest to occur
9. Adequacy of follow up of cohorts	▪ Complete follow-up (all patients accounted for)<br>▪ Still score if small amount (15% or less) not accounted for if they're unlikely to introduce bias or if paper describes what happened to them (e.g. discharged/lost contact etc.)	▪ Follow-up rate less than 85%<br>▪ No description of patients unaccounted for

Table S7: **Modified Newcastle-Ottawa Scale for cross-sectional studies**[59]

	Scoring components (any to score)	Non-scoring components
**Selection (out of 4)**		
1. Representativeness of the sample	▪ Representative cohort of COVID-19 patients	▪ Specific cohort of hospitalised COVID-19 patients (only those with e.g. cancer/IBD/ admitted to ICU etc.)<br>▪ No description
2. Sample size	▪ Justified and satisfactory	▪ Not justified
3. Non-respondents (e.g. excluded patients)	▪ Characteristics of respondents and non-respondents compared (e.g. described basic characteristics of the excluded patients)	▪ No description
4. Ascertainment of exposure	▪ Secure record<br>▪ Structured interview conducted blind to case/ control status	▪ Self-reported<br>▪ Interview not blinded to case/control<br>▪ No description
**Comparability (out of 2)**		
5. Age	▪ Adjusted for age in multivariate model<br>▪ Cohorts matched by age in design	▪ Statements of no difference between groups/ differences were not statistically significant
6. Any other significant risk factor	▪ As above, but for any other significant risk factor (such as gender or comorbidities)	▪ Statements of no difference between groups/ differences were not statistically significant
**Outcome (out of 2)**		
7. Assessment of outcome	▪ Secure record/database<br>▪ Independent assessment (e.g. more than 1 person/record/time/process)	▪ Self-reported<br>▪ No reference to original medical records<br>▪ No description of how outcomes extracted
8. Statistical test	▪ Statistical test(s) appropriate and clearly described, with OR/RR, confidence intervals and p-values presented	▪ Statistical test(s) not appropriate or described

Conversion of Newcastle-Ottawa Scale scores to overall grade of study quality

Score requirements in case series and cohort studies:

- Good-quality: 3 or 4 points in the selection domain AND 1 or 2 points in the comparability domain AND 2 or 3 points in the exposure/outcome domains
- Fair-quality: 2 points in the selection domain AND 1 or 2 points in the comparability domain AND 2 or 3 points in the exposure/outcome domains
- Poor-quality: 0 or 1 points in the selection domain OR 0 points in the comparability domain OR 0 or 1 points in the exposure/outcome domains

Score requirements in cross-sectional studies:

- Good-quality: 3 or 4 points in the selection domain AND 1 or 2 points in the comparability domain AND 1 or 2 points in the outcome domain
- Fair-quality: 2 points in the selection domain AND 1 or 2 points in the comparability domain AND 1 or 2 points in the outcome domain
- Poor-quality: 0 or 1 points in the selection domain OR 0 points in the comparability domain OR 0 points in the outcome domain

SUPPLEMENTARY RESULTS

Table S8: Breakdown of Newcastle-Ottawa Scale grading for included studies

	Selection domains				Comparability domain	Exposure/Outcome domains			Overall quality
	1	2	3	4		1	2	3	
Azar K et al[12]	×	✓	✓	×	✓✓	✓	✓	✓	Fair
Bhargava A et al[13]	✓	✓	✓	✓	✓✓	✓	✓	✓	Good
Bi X et al[14]	✓	✓	✓	✓	✓✓	✓	×	✓	Good
Brenner E et al[15]	×	✓	✓	×	✓✓	×	✓	×	Poor
Buckner F et al[16]	✓	✓	✓	×	×	✓	×	✓	Poor
CDC COVID-19 Response Team[17]	✓	✓	✓	×	×	✓	×	✓	Poor
Chen Q et al[18]	✓	✓	×	×	×	✓	✓	✓	Poor
Chen R et al[19]	✓	✓	✓	✓	✓✓	✓	✓	✓	Good
Chen T et al[20]	✓	✓	×	×	×	✓	✓	✓	Poor
Docherty A et al[21]	✓	✓	✓	✓	✓	✓	×	✓	Good
Feng Y et al[22]	✓	✓	✓	×	✓✓	✓	×	✓	Good
Goyal P et al[23]	✓	✓	×	×	×	✓	✓	×	Poor
Guan W et al[24]	✓	✓	×	×	×	✓	×	✓	Poor
Hu L et al[25]	✓	✓	✓	✓	✓✓	✓	×	✓	Good
Huang C et al[26]	✓	✓	×	×	×	✓	×	✓	Poor
Huang J et al[27]	✓	✓	✓	✓	✓✓	×	✓	✓	Good
Huang R et al[28]	✓	✓	✓	✓	✓	✓	×	✓	Good
Hur K et al[29]	✓	✓	✓	✓	✓✓	✓	×	✓	Good
Inciardi R et al[30]	×	✓	✓	✓	×	✓	×	✓	Poor
Ji D et al[31]	✓	✓	✓	✓	✓✓	✓	×	✓	Good
Kalligeros M et al[32]	✓	✓	✓	✓	✓✓	✓	×	✓	Good
Klang E et al[33]	✓	✓	✓	✓	✓✓	×	✓	✓	Good
Kuderer N et al[34]	×	✓	✓	×	✓✓	✓	×	✓	Fair
Li X et al[35]	✓	✓	✓	✓	✓✓	✓	×	✓	Good
Li Y et al[36]	✓	×	×	×	×	✓	×	✓	Poor
Liu W et al[37]	✓	✓	✓	✓	✓✓	✓	×	✓	Good
Petrilli C et al[39]	✓	✓	×	✓	✓✓	×	✓	✓	Good
Qin C et al[38]	✓	✓	✓	×	×	×	×	×	Poor
Rastrelli G et al[40]	✓	✓	✓	✓	×	✓	×	✓	Poor
Shi Q et al[41]	✓	✓	✓	✓	✓✓	✓	×	✓	Good
Shi Y et al[42]	✓	✓	✓	×	✓✓	✓	×	✓	Good
Sun D et al[43]	✓	✓	✓	✓	✓	✓	✓	✓	Good
Toussie D et al[44]	×	✓	×	✓	✓✓	✓	×	✓	Fair
Wan S et al[45]	✓	✓	×	×	×	×	×	✓	Poor
Wang K et al[46]	✓	✓	✓	✓	✓✓	×	×	✓	Poor
Wang R et al[47]	✓	✓	×	×	×	✓	✓	✓	Poor
Yang X et al[48]	✓	✓	✓	×	×	✓	×	✓	Poor
Yao Q et al[49]	✓	✓	✓	✓	✓	✓	✓	✓	Good
Yu Q et al[50]	✓	✓	✓	✓	✓✓	✓	✓	✓	Good
Yu T et al[51]	✓	✓	✓	✓	✓✓	✓	✓	N/A	Good
Yu X, Sun S et al[52]	×	✓	×	×	×	×	×	✓	Poor
Yu X, Sun X et al[53]	×	✓	✓	×	✓✓	✓	×	✓	Fair
Zhang J et al[54]	✓	✓	×	×	×	×	✓	✓	Poor
Zhang R et al[55]	✓	✓	×	×	✓✓	✓	×	✓	Fair
Zheng K et al[56]	✓	✓	×	×	✓✓	×	✓	✓	Fair
Zheng Y et al[57]	✓	✓	×	×	×	×	×	✓	Poor
Zhou F et al[58]	✓	✓	✓	✓	✓✓	✓	✓	✓	Good

Figure S1: **Funnel plots for the effect of current smoking on severe COVID-19 (A), severe or critical COVID-19 (B), and mortality (C)**

A

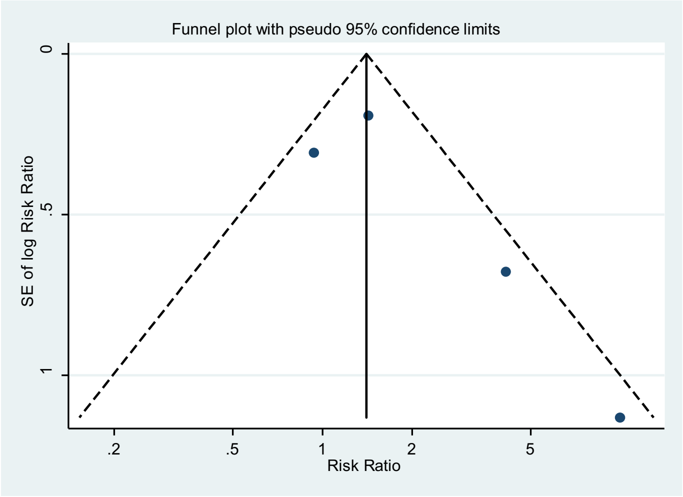

B

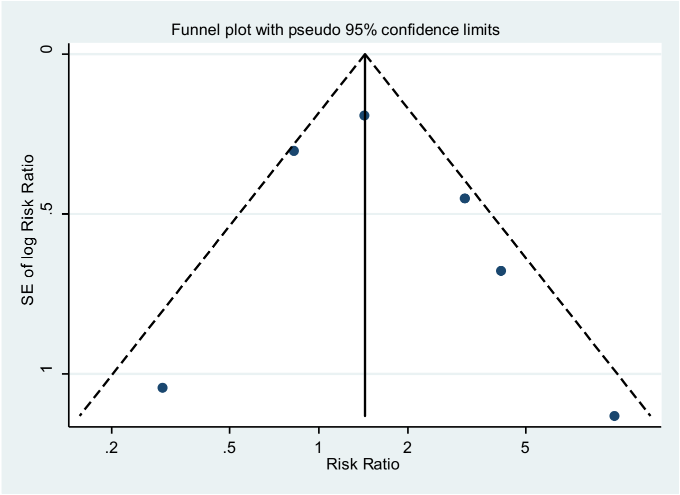

C

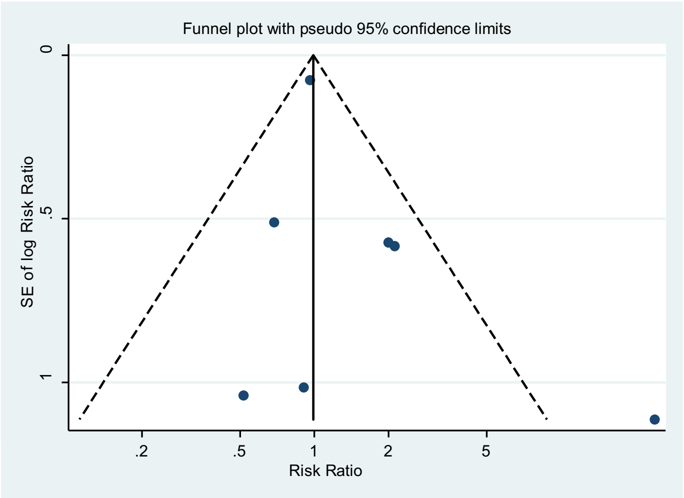

Figure S2: **Forest and funnel plots for the effect of current smoking on disease progression (A), ICU admission (B), and mechanical ventilation requirement (C)**

A. Disease progression

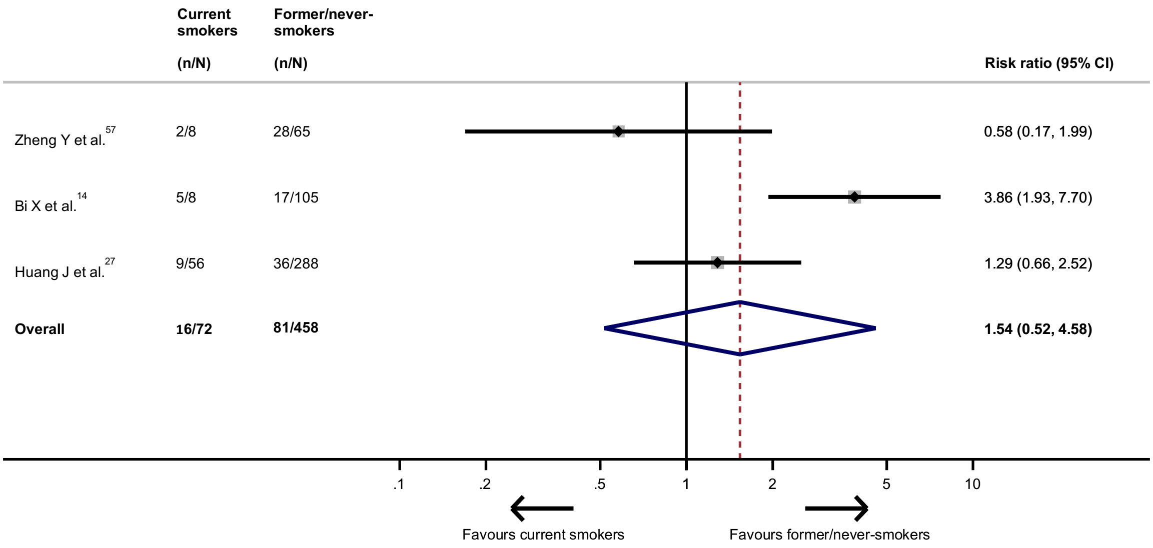

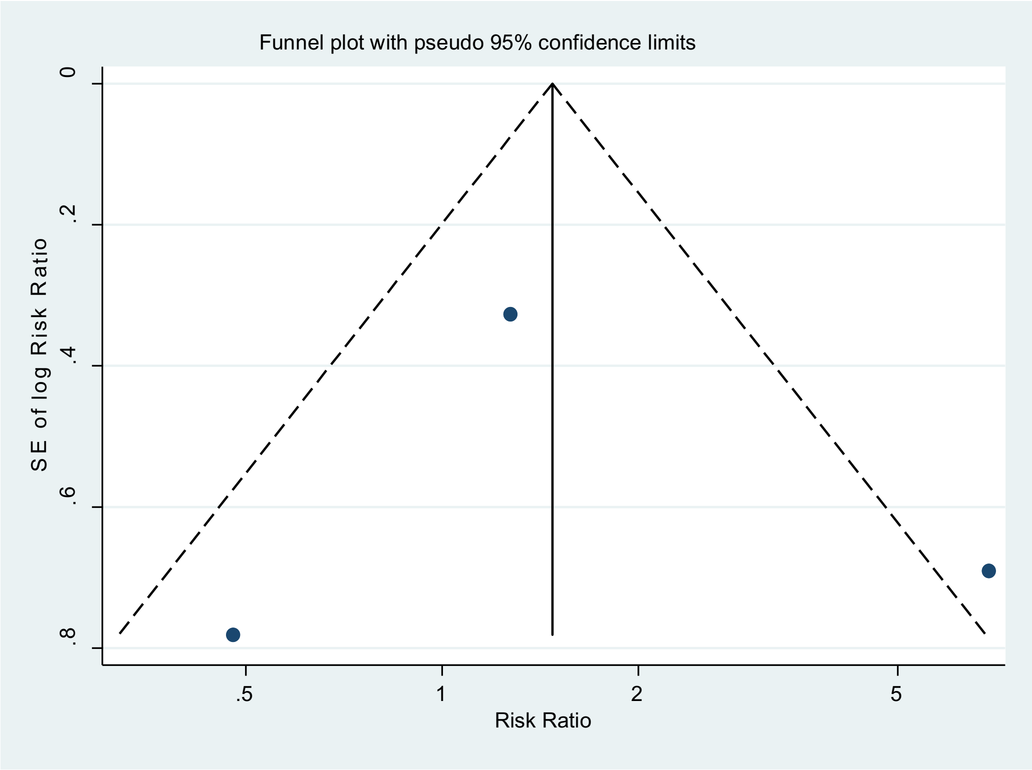

B. ICU admission

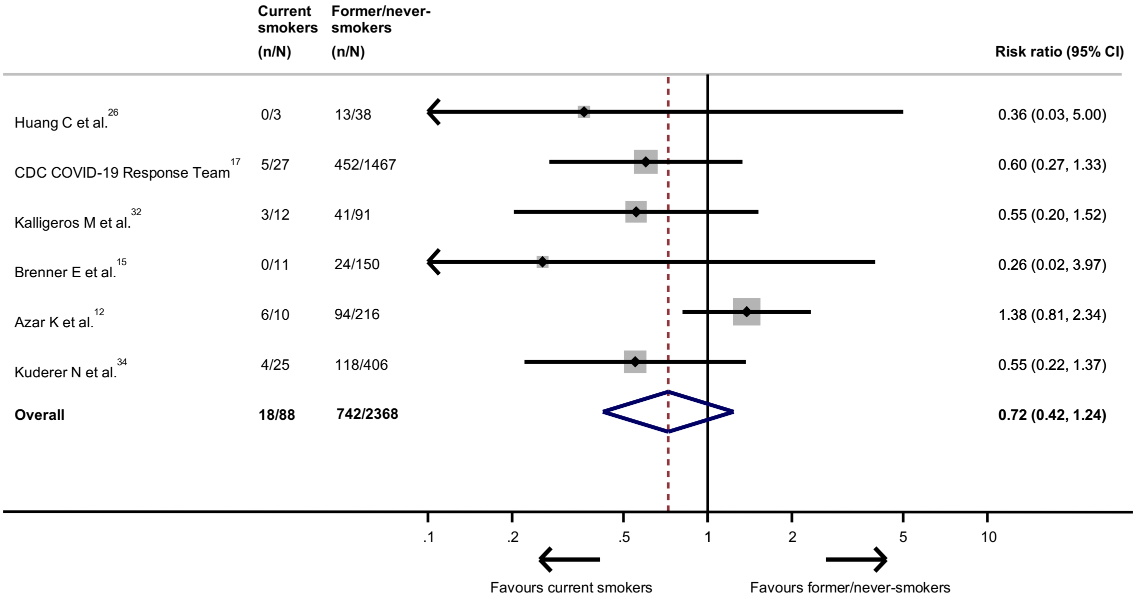

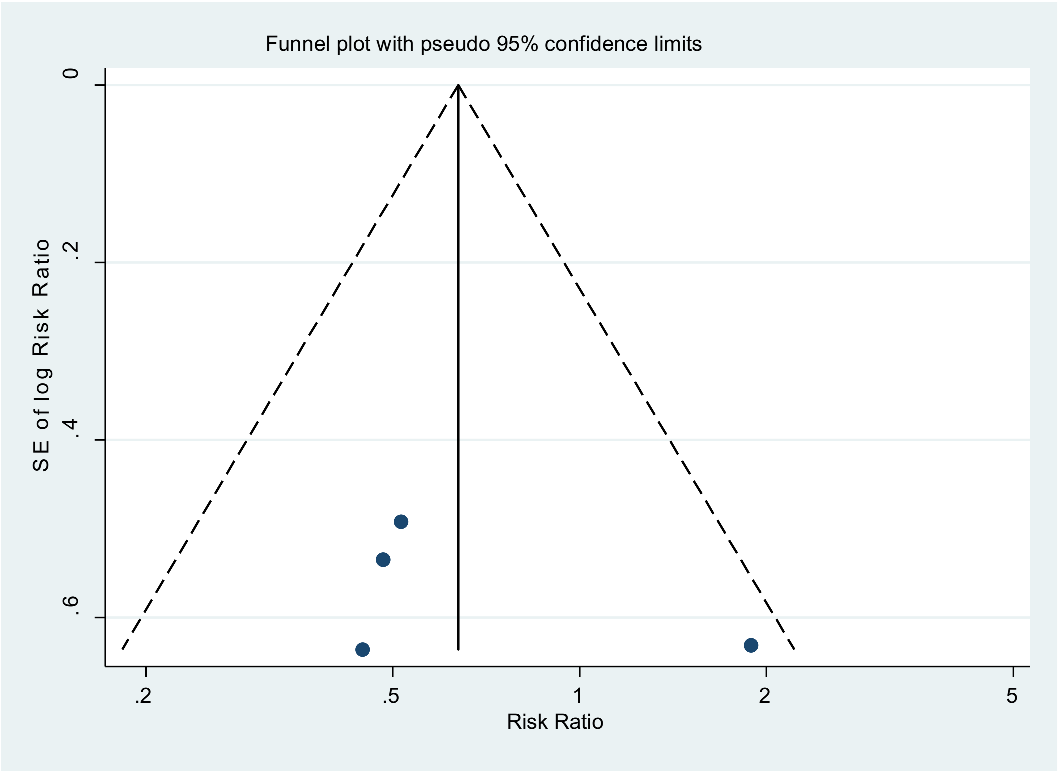

C. Mechanical ventilation requirement

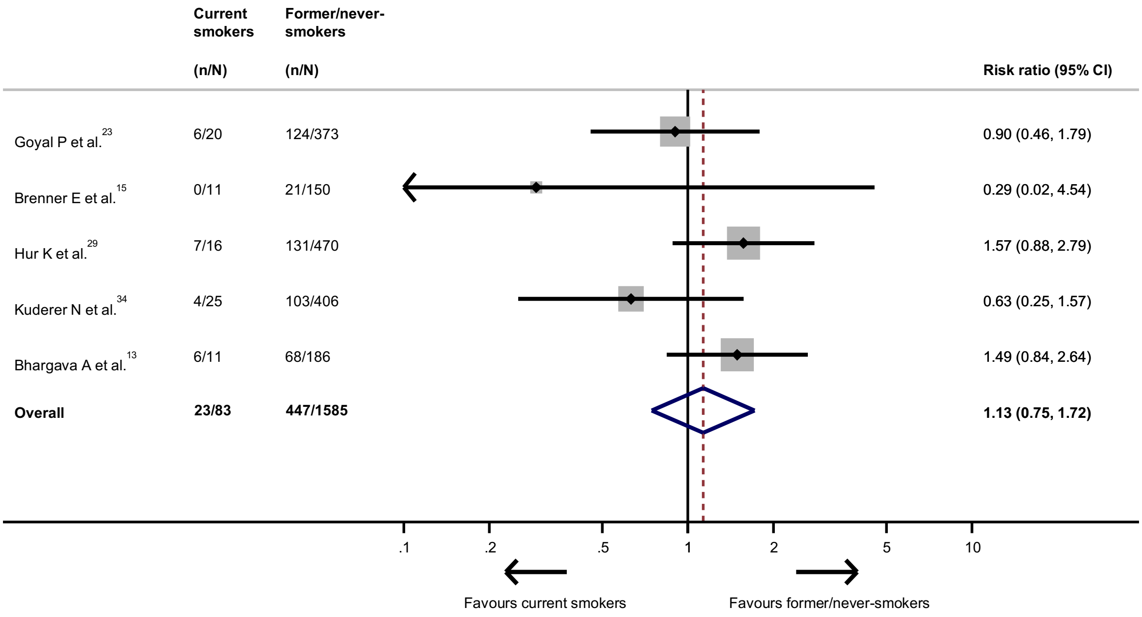

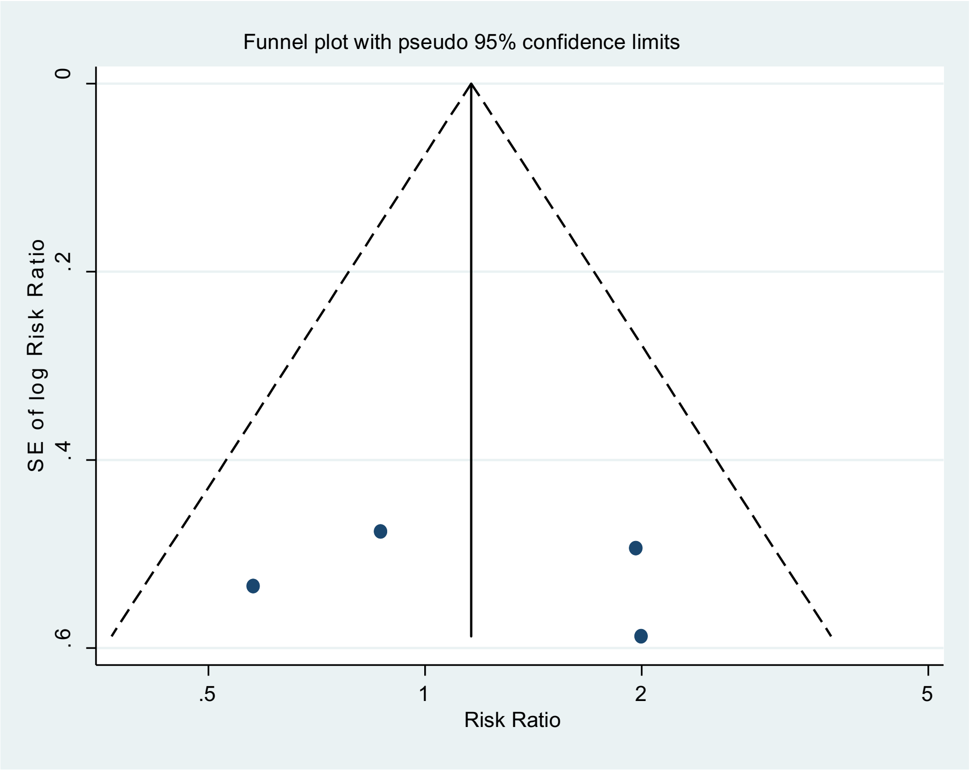

***Table S9:* Sensitivity analyses of the effect of current smoking**

	**No. studies**	**Current smokers (n/N)**	**Former/never-smokers (n/N)**	**Pooled risk ratio (95% CI)**	**p-value**	**I^2 (%)**
**Studies using the COVID-19-specific criteria only**						
Severe disease	2	7/10	70/196	2.24 (1.45-3.44)	<0.0001	0
Severe or critical disease	5	21/41	151/545	2.43 (1.34-4.40)	0.003	74
**Good-quality studies only**						
Severe disease	2	19/45	227/607	1.79 (0.46-7.02)	0.401	92
Severe or critical disease	2	21/45	269/607	1.73 (0.42-7.14)	0.448	93
Mortality	3	222/867	3513/13616	2.28 (0.70-7.42)	0.172	93

Sensitivity analyses were not performed for outcomes with only one study following restrictions.

Figure S3: **Forest plot showing the effect of current smoking on mortality by country**

	Current smokers (n/N)	Former/never-smokers (n/N)	Risk ratio (95% CI)
**China**			
Chen T et al.[20]	7/12	106/262	1.44 (0.87, 2.38)
Zhou F et al.[58]	5/11	49/180	1.67 (0.84, 3.33)
Wang K et al.[46]	4/41	70/503	0.70 (0.27, 1.82)
Yao Q et al.[49]	3/4	9/104	8.67 (3.73, 20.13)
Subtotal	19/68	234/1049	1.96 (0.76, 5.03)
**International**			
Brenner E et al.[15]	1/11	15/150	0.91 (0.13, 6.26)
Subtotal	1/11	15/150	0.91 (0.13, 6.26)
**UK**			
Docherty A et al.[21]	214/852	3455/13332	0.97 (0.86, 1.09)
Subtotal	214/852	3455/13332	0.97 (0.86, 1.09)
**USA**			
Azar K et al.[12]	1/10	39/216	0.55 (0.08, 3.63)
Subtotal	1/10	39/216	0.55 (0.08, 3.63)
**Overall**	**235/941**	**3743/14747**	**1.46 (0.83, 2.60)**

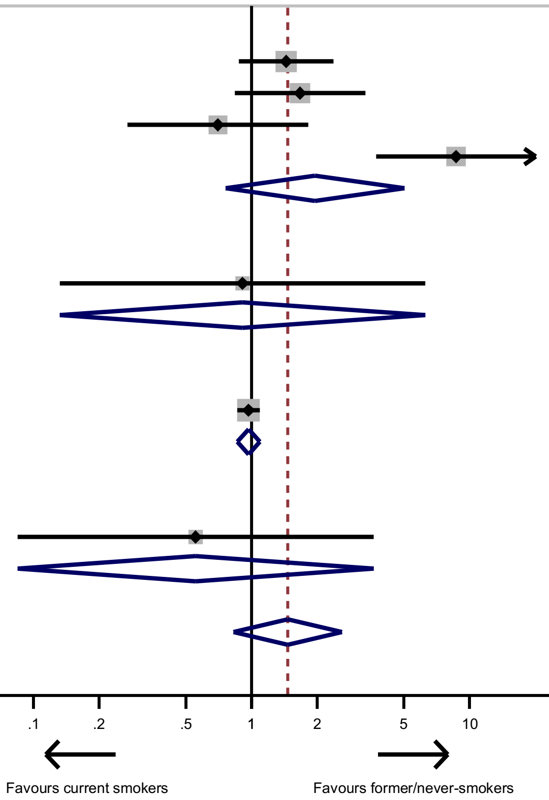

Figure S4: **Funnel plots for the effect of a smoking history on severe COVID-19 (A), severe or critical COVID-19 (B), and mortality (C)**

A

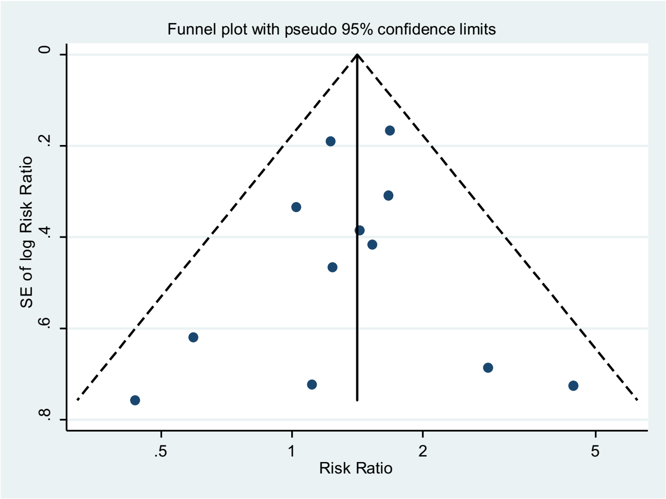

B

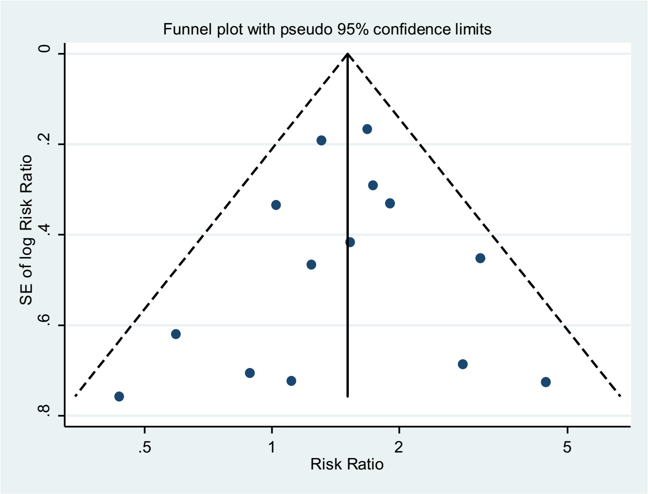

C

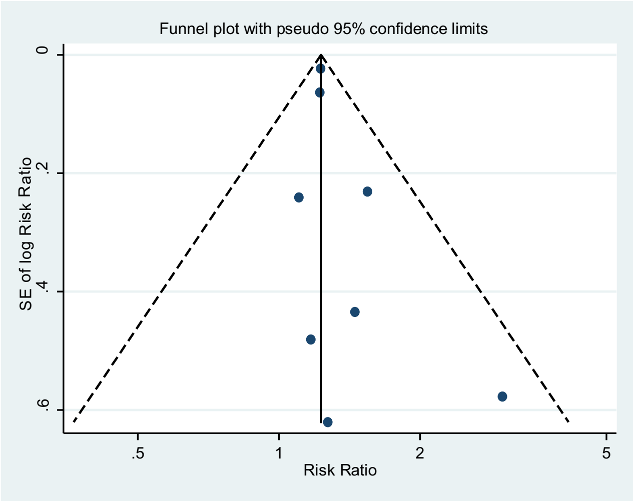

Figure S5: **Forest and funnel plots for the effect of a smoking history on critical COVID-19 alone (A), disease progression (B), ICU admission (C), and mechanical ventilation requirement (D)**

A. Critical COVID-19 alone

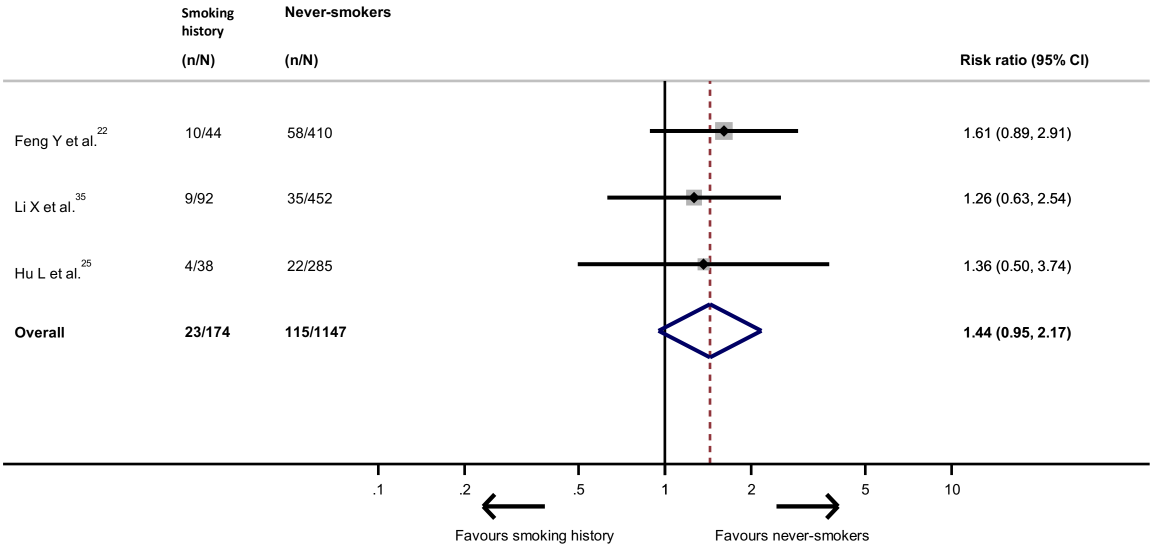

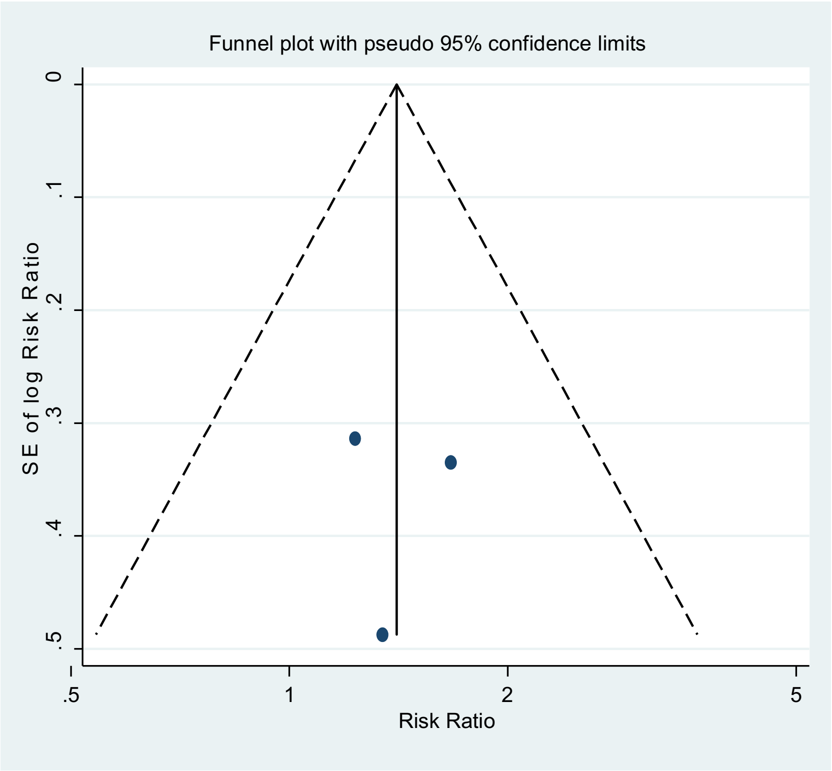

B. Disease progression

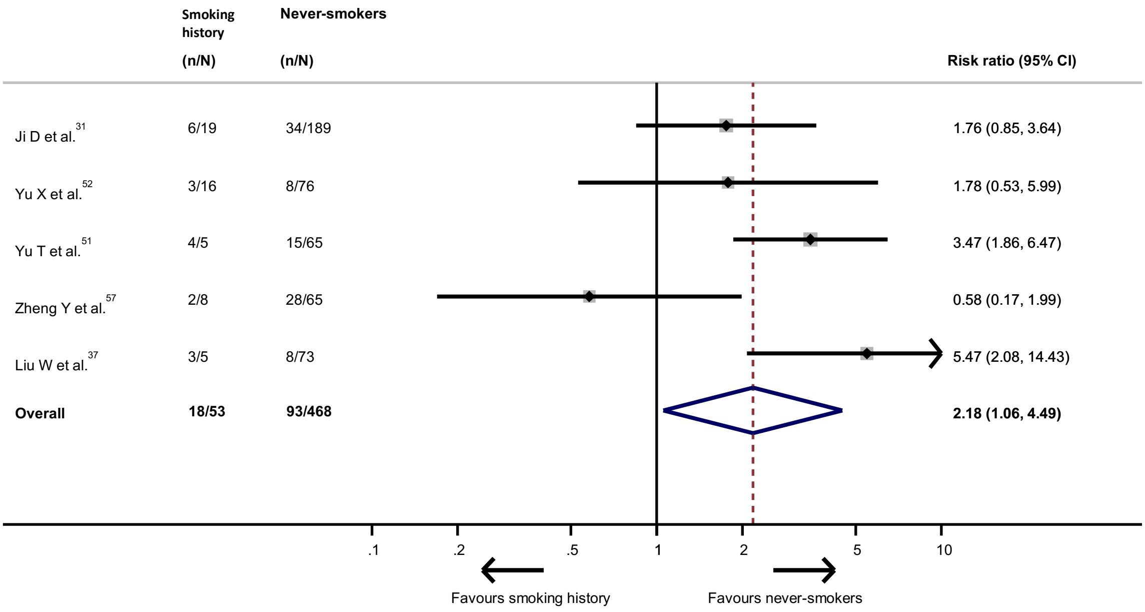

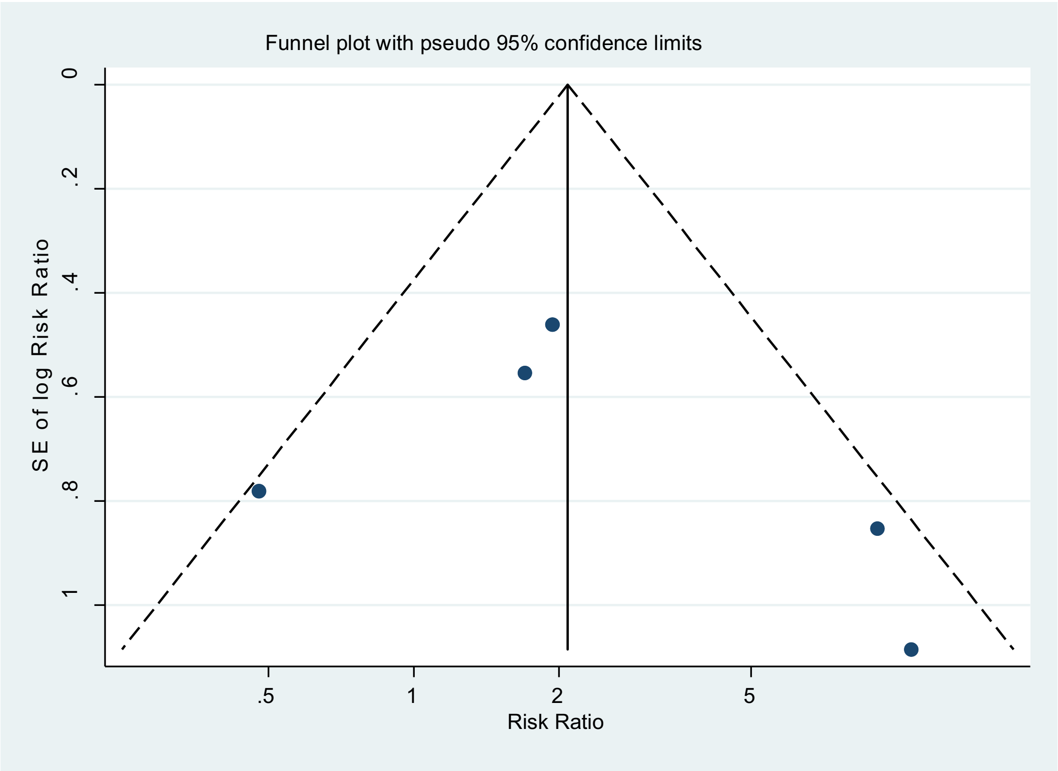

Disease progression was the only outcome in which Harbord's test implied publication bias (p=0.049).

C. ICU admission

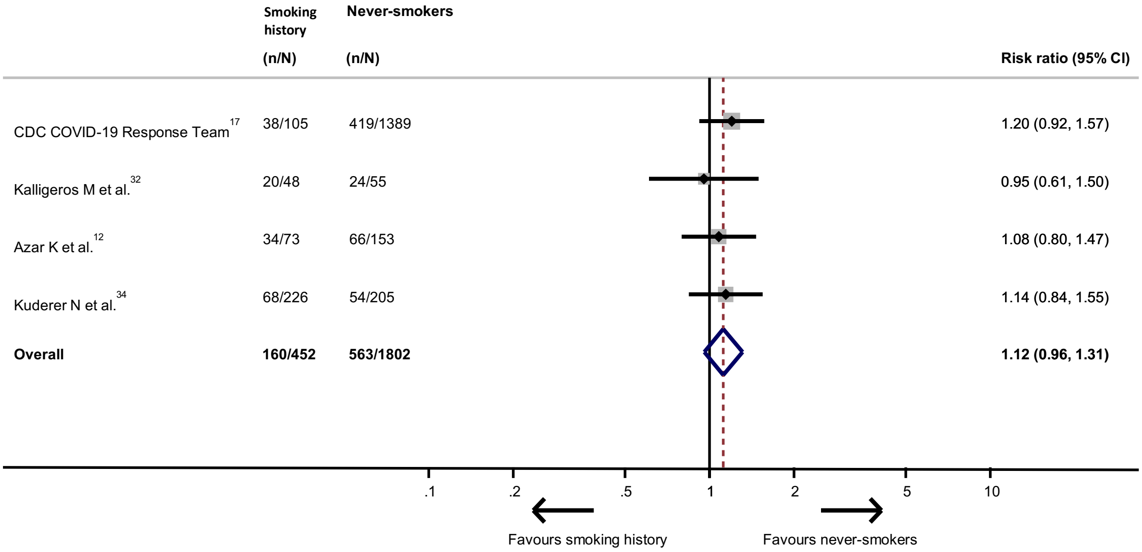

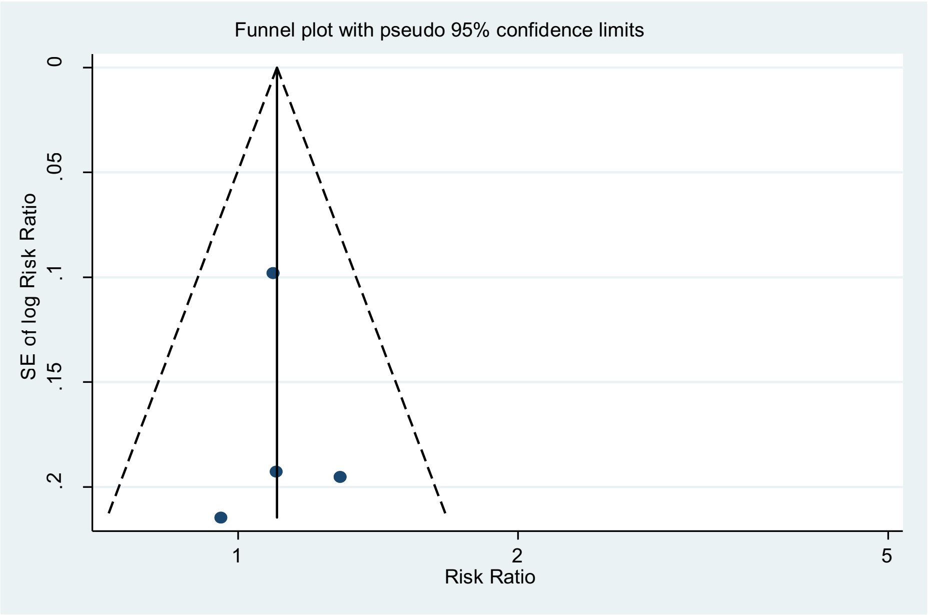

D. Mechanical ventilation requirement

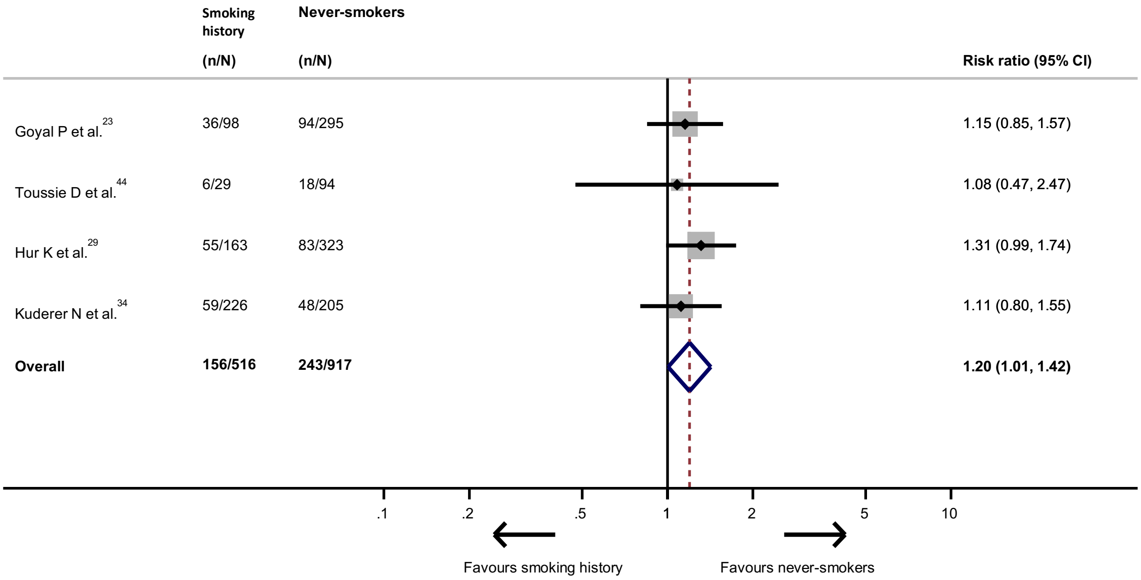

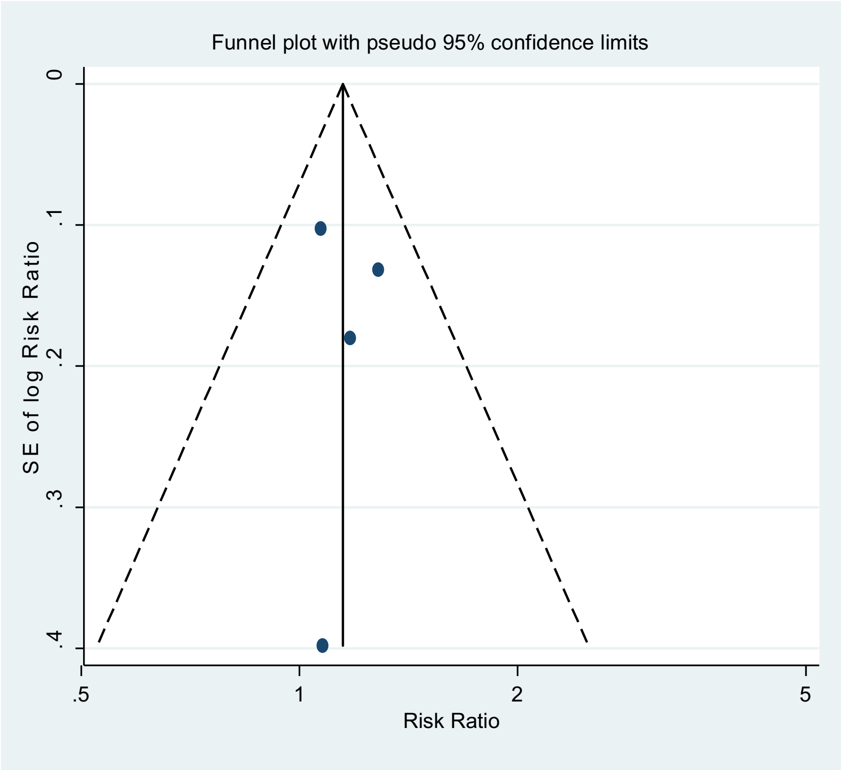

Table S10: **Sensitivity analyses of the effect of a smoking history**

	No. studies	Smoking history (n/N)	Never-smokers (n/N)	Pooled risk ratio (95% CI)	p-value	I^2 (%)
**Studies using the COVID-19-specific criteria only**						
Severe disease	7	48/136	570/1605	1.33 (0.97-1.81)	0.072	30
Critical disease	2	14/82	80/695	1.54 (0.92-2.57)	0.099	0
Severe or critical disease	10	83/190	724/2024	1.41 (1.16-1.70)	<0.0001	28
**Good-quality studies only**						
Severe disease	5	79/230	413/1767	1.24 (1.04-1.49)	0.017	0
Critical disease	3	23/174	115/1147	1.44 (0.95-2.17)	0.085	0
Severe or critical disease	6	114/242	561/1812	1.29 (1.15-1.45)	<0.0001	0
Disease progression	3	13/29	57/327	3.08 (1.62-5.87)	0.001	54
Mortality	4	1873/6136	3029/13350	1.26 (1.20-1.32)	<0.0001	0

Sensitivity analyses were not performed for outcomes with only one study following restrictions.

Figure S6: **Forest plot showing the effect of a smoking history on mortality by country**

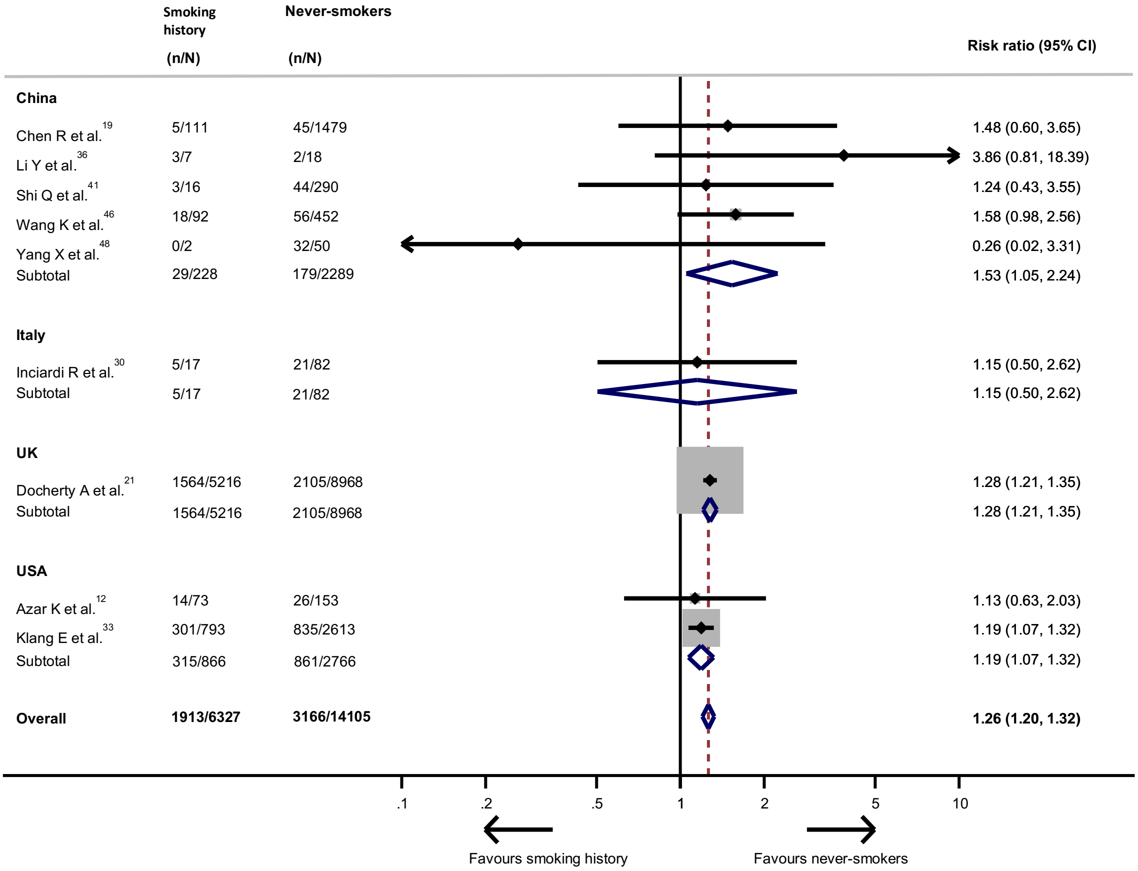

SUPPLEMENTARY REFERENCES

1. Karanasos A, Aznaouridis K, Latsios G et al. Impact of Smoking Status on Disease Severity and Mortality of Hospitalized Patients With COVID-19 Infection: *Nicotine Tob Res* 2020; published online June 20. doi: 10.1093/ntr/ntaa107.
2. Patanavanich R, Glantz S. Smoking Is Associated With COVID-19 Progression: A Meta-Analysis. *Nicotine Tob Res* 2020; published online May 13. doi: 10.1093/ntr/ntaa082.
3. Zheng Z, Peng F, Xu B et al. Risk factors of critical & mortal COVID-19 cases: A systematic literature review and meta-analysis. *J Infect* 2020; published online April 23. doi: 10.1016/j.jinf.2020.04.021.
4. Zhao Q, Meng M, Kumar R et al. The Impact of COPD and Smoking History on the Severity of COVID-19: A Systemic Review and Meta-Analysis. *J Med Virol* 2020; published online April 15. doi: 10.1002/jmv.25889.
5. Lippi G, Henry BM. Active smoking is not associated with severity of coronavirus disease 2019 (COVID-19). *Eur J Intern Med* 2020; **75**: 107–8.
6. Vardavas CI, Nikitara K. COVID-19 and smoking: A systematic review of the evidence. *Tob Induc Dis* 2020; **18**: 20.
7. Shea BJ, Reeves BC, Wells G et al**.** AMSTAR 2: A Critical Appraisal Tool for Systematic Reviews That Include Randomised or Non-Randomised Studies of Healthcare Interventions, or Both. *BMJ* 2017; **358**: j4008.
8. Khajuria A, Charles W, Sklavounos A, Reddy R. The effects of smoking on COVID-19 severity: a systematic review and meta-analysis. April 27 2020. https://www.crd.york.ac.uk/prospero/display_record.php?RecordID=180920 (accessed July 4 2020).
9. Moher D, Liberati A, Tetzlaff J, Altman, DG, PRISMA Group. Preferred Reporting Items for Systematic Reviews and Meta-Analyses: The PRISMA Statement. *BMJ* 2009; **339**: b2535.
10. National Health Commission & National Administration of Traditional Chinese Medicine. Diagnosis and Treatment Protocol for Novel Coronavirus Pneumonia (Trial Version 7). *Chin Med J (Engl)* 2020; **133**: 1087–1095.
11. Metlay JP, Waterer GW, Long AC et al. Diagnosis and Treatment of Adults With Community-acquired Pneumonia. An Official Clinical Practice Guideline of the American Thoracic Society and Infectious Diseases Society of America. *Am J Respir Crit Care Med* 2020; **200**: e45-67.
12. Azar KMJ, Shen Z, Romanelli RJ et al. Disparities In Outcomes Among COVID-19 Patients In A Large Health Care System In California. *Health Aff (Millwood)* 2020; published online May 21. doi: 10.1377/hlthaff.2020.00598.
13. Bhargava A, Fukushima EA, Levine M et al. Predictors for Severe COVID-19 Infection. *Clin Infect Dis* 2020; published online May 30. doi: 10.1093/cid/ciaa674.
14. Bi X, Su Z, Yan H et al. Prediction of severe illness due to COVID-19 based on an analysis of initial Fibrinogen to Albumin Ratio and Platelet count. *Platelets* 2020; published online May 5. doi: 10.1080/09537104.2020.1760230.
15. Brenner EJ, Ungaro RC, Gearry RB et al. Corticosteroids, but Not TNF Antagonists, Are Associated With Adverse COVID-19 Outcomes in Patients With Inflammatory Bowel Diseases: Results From an International Registry. *Gastroenterology* 2020; published online May 18. doi: 10.1053/j.gastro.2020.05.032.
16. Buckner FS, McCulloch DJ, Atluri V et al. Clinical Features and Outcomes of 105 Hospitalized Patients With COVID-19 in Seattle, Washington. *Clin Infect Dis* 2020; published online May 22. doi: 10.1093/cid/ciaa632.
17. CDC COVID-19 Response Team. Preliminary Estimates of the Prevalence of Selected Underlying Health Conditions Among Patients With Coronavirus Disease 2019 - United States, February 12-March 28, 2020. *MMWR Morb Mortal Wkly Rep* 2020; **69**: 283–386.
18. Chen Q, Zheng Z, Zhang C et al. Clinical characteristics of 145 patients with corona virus disease 2019 (COVID-19) in Taizhou, Zhejiang, China. *Infection* 2020; published online April 28. doi: 10.1007/s15010-020-01432-5.
19. Chen R, Liang W, Jiang M et al. Risk Factors of Fatal Outcome in Hospitalized Subjects With Coronavirus Disease 2019 From a Nationwide Analysis in China. *Chest* 2020; **158**: 97–105.
20. Chen T, Wu D, Chen H et al. Clinical Characteristics of 113 Deceased Patients With Coronavirus Disease 2019: Retrospective Study. *BMJ* 2020; **368**: m1091.
21. Docherty AB, Harrison EM, Green CA et al. Features of 20 133 UK Patients in Hospital With covid-19 Using the ISARIC WHO Clinical Characterisation Protocol: Prospective Observational Cohort Study. *BMJ* 2020; **369**: m1985.
22. Feng Y, Ling Y, Bai T et al. COVID-19 With Different Severities: A Multicenter Study of Clinical Features. *Am J Respir Crit Care Med* 2020; **201**: 1380–1388.

23. Goyal P, Choi JJ, Pinheiro LC et al. Clinical Characteristics of Covid-19 in New York City. *N Engl J Med* 2020; **382**: 2372–2374.
24. Guan WJ, Ni ZY, Hu Y et al. Clinical Characteristics of Coronavirus Disease 2019 in China. *N Engl J Med* 2020; **382**: 1708–1720.
25. Hu L, Chen S, Fu Y et al. Risk Factors Associated With Clinical Outcomes in 323 COVID-19 Hospitalized Patients in Wuhan, China. *Clin Infect Dis* 2020; published online May 23. doi: 10.1093/cid/ciaa539.
26. Huang C, Wang Y, Li X et al. Clinical Features of Patients Infected With 2019 Novel Coronavirus in Wuhan, China. *Lancet* 2020; **395**: 497–506.
27. Huang J, Cheng A, Lin S, Zhu Y, Chen G. Individualized Prediction Nomograms for Disease Progression in Mild COVID-19. *J Med Virol* 2020; published online May 5. doi: 10.1002/jmv.25969.
28. Huang R, Zhu L, Xue L et al. Clinical Findings of Patients With Coronavirus Disease 2019 in Jiangsu Province, China: A Retrospective, Multi-Center Study. PLoS Negl Trop Dis 2020; **14**: e0008280.
29. Hur K, Price CPE, Gray EL et al. Factors Associated With Intubation and Prolonged Intubation in Hospitalized Patients With COVID-19. *Otolaryngol Head Neck Surg* 2020; **163**: 170–178.
30. Inciardi RM, Adamo M, Lupi L et al. Characteristics and Outcomes of Patients Hospitalized for COVID-19 and Cardiac Disease in Northern Italy. *Eur Heart J* 2020; **41**: 1821–1829.
31. Ji D, Zhang D, Xu J et al. Prediction for Progression Risk in Patients With COVID-19 Pneumonia: The CALL Score. *Clin Infect Dis* 2020; published online April 9. doi: 10.1093/cid/ciaa414.
32. Kalligeros M, Shehadeh F, Mylona EK et al. Association of Obesity With Disease Severity Among Patients With Coronavirus Disease 2019. *Obesity (Silver Spring)* 2020; **28**: 1200–1204.
33. Klang E, Kassim G, Soffer S, Freeman R, Levin MA, Reich DL. Morbid Obesity as an Independent Risk Factor for COVID-19 Mortality in Hospitalized Patients Younger Than 50. *Obesity (Silver Spring)* 2020; published online May 23. doi: 10.1002/oby.22913.
34. Kuderer NM, Choueiri TK, Shah DP, et al. Clinical impact of COVID-19 on patients with cancer (CCC19): a cohort study. *Lancet* 2020; **395:** 1907–1918.
35. Li X, Xu S, Yu M, et al. Risk factors for severity and mortality in adult COVID-19 inpatients in Wuhan. *J Allergy Clin Immunol* 2020; published online April 12. doi:10.1016/j.jaci.2020.04.006.
36. Li YK, Peng S, Li LQ, et al. Clinical and Transmission Characteristics of Covid-19 - A Retrospective Study of 25 Cases from a Single Thoracic Surgery Department. *Curr Med Sci* 2020; **40:** 295–300.
37. Liu W, Tao ZW, Wang L, et al. Analysis of factors associated with disease outcomes in hospitalized patients with 2019 novel coronavirus disease. *Chin Med J (Engl)* 2020; **133:** 1032–1038.
38. Qin C, Zhou L, Hu Z, et al. Dysregulation of immune response in patients with COVID-19 in Wuhan, China. *Clin Infect Dis* 2020; published online March 12. doi:10.1093/cid/ciaa248.
39. Petrilli CM, Jones SA, Yang J, et al. Factors associated with hospital admission and critical illness among 5279 people with coronavirus disease 2019 in New York City: prospective cohort study. *BMJ* 2020; **369:** m1966.
40. Rastrelli G, Di Stasi V, Inglese F, et al. Low testosterone levels predict clinical adverse outcomes in SARS-CoV-2 pneumonia patients. *Andrology* 2020; published online May 20. doi:10.1111/andr.12821.
41. Shi Q, Zhang X, Jiang F, et al. Clinical Characteristics and Risk Factors for Mortality of COVID-19 Patients With Diabetes in Wuhan, China: A Two-Center, Retrospective Study. *Diabetes Care* 2020; **43:** 1382–1391.
42. Shi Y, Yu X, Zhao H, Wang H, Zhao R, Sheng J. Host susceptibility to severe COVID-19 and establishment of a host risk score: findings of 487 cases outside Wuhan. *Crit Care* 2020; **24:** 108.
43. Sun DW, Zhang D, Tian RH, et al. The underlying changes and predicting role of peripheral blood inflammatory cells in severe COVID-19 patients: A sentinel? *Clin Chim Acta* 2020; published online May 14. doi:10.1016/j.cca.2020.05.027.
44. Toussie D, Voutsinas N, Finkelstein M, et al. Clinical and Chest Radiography Features Determine Patient Outcomes In Young and Middle Age Adults with COVID-19. *Radiology* 2020; published online May 14. doi:10.1148/radiol.2020201754.
45. Wan S, Xiang Y, Fang W, et al. Clinical features and treatment of COVID-19 patients in northeast Chongqing. *J Med Virol* 2020; **92:** 797–806.
46. Wang K, Zhang Z, Yu M, Tao Y, Xie M. 15-day mortality and associated risk factors for hospitalized patients with COVID-19 in Wuhan, China: an ambispective observational cohort study. *Intensive Care Med* 2020; published online Apr 23. doi:10.1007/s00134-020-06047-w.
47. Wang R, Pan M, Zhang X, et al. Epidemiological and clinical features of 125 Hospitalized Patients with COVID-19 in Fuyang, Anhui, China. *Int J Infect Dis* 2020; **95:** 421–428.
48. Yang X, Yu Y, Xu J, et al. Clinical course and outcomes of critically ill patients with SARS-CoV-2 pneumonia in Wuhan, China: a single-centered, retrospective, observational study. *Lancet Respir Med* 2020; **8:** 475–481.

49. Yao Q, Wang P, Wang X, et al. A retrospective study of risk factors for severe acute respiratory syndrome coronavirus 2 infections in hospitalized adult patients. *Pol Arch Intern Med* 2020; **130:** 390–399.
50. Yu Q, Wang Y, Huang S, et al. Multicenter cohort study demonstrates more consolidation in upper lungs on initial CT increases the risk of adverse clinical outcome in COVID-19 patients. *Theranostics* 2020; **10:** 5641–5648.
51. Yu T, Cai S, Zheng Z, et al. Association Between Clinical Manifestations and Prognosis in Patients with COVID-19. *Clin Ther* 2020; **42:** 964–972.
52. Yu X, Sun S, Shi Y, Wang H, Zhao R, Sheng J. SARS-CoV-2 viral load in sputum correlates with risk of COVID-19 progression. *Crit Care* 2020; **24:** 170.
53. Yu X, Sun X, Cui P, et al. Epidemiological and clinical characteristics of 333 confirmed cases with coronavirus disease 2019 in Shanghai, China. *Transbound Emerg Dis* 2020; published online Apr 29. doi:10.1111/tbed.13604.
54. Zhang JJ, Dong X, Cao YY, et al. Clinical characteristics of 140 patients infected with SARS-CoV-2 in Wuhan, China. *Allergy* 2020; published online Feb 19. doi:10.1111/all.14238.
55. Zhang R, Ouyang H, Fu L, et al. CT features of SARS-CoV-2 pneumonia according to clinical presentation: a retrospective analysis of 120 consecutive patients from Wuhan city. *Eur Radiol* 2020; published online Apr 11. doi:10.1007/s00330-020-06854-1.
56. Zheng KI, Gao F, Wang XB, et al. Letter to the Editor: Obesity as a risk factor for greater severity of COVID-19 in patients with metabolic associated fatty liver disease. *Metabolism* 2020; **108:** 154244.
57. Zheng Y, Xiong C, Liu Y, et al. Epidemiological and clinical characteristics analysis of COVID-19 in the surrounding areas of Wuhan, Hubei Province in 2020. *Pharmacol Res* 2020; **157:** 104821.
58. Zhou F, Yu T, Du R et al. Clinical Course and Risk Factors for Mortality of Adult Inpatients With COVID-19 in Wuhan, China: A Retrospective Cohort Study. *Lancet* 2020; **395**: 1054–1062.
59. GA Wells BS, D O'Connell, Peterson, V Welch, M Losos, P Tugwell. The Newcastle-Ottawa Scale (NOS) for assessing the quality of nonrandomised studies in meta-analyses. 2019. http://www.ohri.ca/programs/clinical_epidemiology/oxford.asp (accessed July 4 2020).
